# Contextual factors influencing bubble continuous positive airway pressure implementation for paediatric respiratory distress in low-income and middle-income countries: a realist review

**DOI:** 10.1016/S2214-109X(24)00453-4

**Published:** 2024-12-12

**Authors:** Nadir Ijaz, Marie Nader, Matthew Ponticiello, Ashlee J Vance, Brittney J van de Water, Melissa C Funaro, Qalab Abbas, John Adabie Appiah, Mohammod Jobayer Chisti, Walter Commerell, Suiyven Elvis Dzelamunyuy, Rudimar Martinez Fernandez, Anjelica L Gonzalez, Cintia Johnston, Evance Luckson Kaiwe, Manjinder Kaur, Hans-Joerg Lang, Eric D McCollum, José Marcos González Moraga, Jayashree Muralidharan, Kelsey Renning, Herng Lee Tan, Laura Alejandra Vélez Ruiz Gaitán, Sebastián González-Dambrauskas, Patrick T Wilson, Brenda M Morrow, J Lucian Davis

**Affiliations:** Yale School of Medicine, New Haven, CT, USA (N Ijaz MD MHS, M Nader MD, M Ponticiello BS, J L Davis MD MAS); Yale National Clinician Scholars Program, New Haven, CT, USA (N Ijaz); Henry Ford Health, Detroit, MI, USA (A J Vance PhD RN); Boston College, Chestnut Hill, PA, USA (B J van de Water PhD CPNP); Harvey Cushing/John Hay Whitney Medical Library, Yale University, New Haven, CT, USA (M C Funaro MS MLS); Department of Paediatrics and Child Health, Aga Khan University, Karachi, Pakistan (Q Abbas MBBS); Paediatric Intensive Care Unit, Komfo Anokye Teaching Hospital, Kumasi, Ghana (J Adabie Appiah MD FWACP); International Centre for Diarrhoeal Disease Research, Dhaka, Bangladesh (M J Chisti MBBS PhD); Technische Hochschule Ulm (THU), Ulm, Germany (Prof W Commerell PhD); Nkwen District Hospital, Bamenda, Cameroon (S Elvis Dzelamunyuy MSN); KK Women’s and Children’s Hospital, Singapore (R Martinez Fernandez RRT, H L Tan MSc RRT-NPS); Department of Biomedical Engineering, Yale University, New Haven, CT, USA (Prof A L Gonzalez PhD); Postgraduate Program in Pediatrics, Faculty of Medicine of the University of São Paulo (FMUSP), São Paulo, Brazil (Prof C Johnston RRT PhD); Mercy James Centre for Paediatric Surgery and Intensive Care Unit, Queen Elizabeth Central Hospital, Blantyre, Malawi (E Luckson Kaiwe BSN); Kamuzu University of Health Sciences, Blantyre, Malawi (E Luckson Kaiwe); Advanced Pediatrics Centre, Post Graduate Institute of Medical Education & Research, Chandigarh, India (M Kaur MSN); Heidelberg Institute for Global Health, Heidelberg, Germany (Prof H-J Lang MD PhD); Alliance for International Medical Action, Dakar, Senegal (Prof H-J Lang); Global Program in Pediatric Respiratory Sciences, Eudowood Division of Pediatric Respiratory Sciences, Johns Hopkins University School of Medicine, Baltimore, MD, USA (E D McCollum MD MPH); Hospital Regional del Libertador Bernardo O’Higgins, Rancagua, Chile (J Marcos González Moraga MD); Advanced Pediatrics Centre, Post Graduate Institute of Medical Education & Research, Chandigarh, India (Prof J Muralidharan MD); Seed Global Health, Blantyre, Malawi (K Renning DNP CPNP-PC); WHO, Geneva, Switzerland (L Alejandra Vélez Ruiz Gaitán MSc); Red Colaborativa Pediátrica de Latinoamérica (LARed Network), Montevideo, Uruguay (S González-Dambrauskas MD); Departamento de Pediatría y Unidad de Cuidados Intensivos de Niños del Centro Hospitalario Pereira Rossell, Facultad de Medicina, Universidad de la República, Montevideo, Uruguay (S González-Dambrauskas); Section of Pediatric Critical Care Medicine, University of Colorado School of Medicine, Aurora, CO, USA (P T Wilson MD MPH); Department of Paediatrics, University of Cape Town, Cape Town, South Africa (Prof B M Morrow PhD); Yale School of Public Health, New Haven, CT, USA (J L Davis)

## Abstract

**Background:**

Bubble continuous positive airway pressure (bCPAP) is a low-cost, non-invasive respiratory support therapy for children with respiratory distress, but its effectiveness is dependent on the context. We aimed to understand contextual factors influencing bCPAP implementation for children aged 1–59 months in low-income and middle-income countries (LMICs) and to develop a theory explaining how these factors influence implementation outcomes.

**Methods:**

In this realist review, we generated an initial programme theory comprising candidate context–mechanism–outcome configurations (CMOCs) via review of key references and team discussion. On July 25, 2023, we conducted a search for peer-reviewed and grey literature, without date restrictions, describing bCPAP use for paediatric respiratory distress in LMICs. We included references describing related contexts, mechanisms, or outcomes. We coded statements from the literature supporting each CMOC, iteratively revising and adding CMOCs using inductive and deductive logic. We assembled an international, interdisciplinary panel of 22 bCPAP stakeholders to refine CMOCs using iterative surveys, focus groups, and interviews until we reached thematic saturation. This realist review is registered with PROSPERO (CRD42023403584).

**Findings:**

Of 1640 peer-reviewed references and eight grey literature references retrieved, 38 peer-reviewed articles and two grey literature documents were deemed eligible for inclusion after removal of duplicates and screening. After four rounds of expert surveys and three focus groups, we identified 18 CMOCs. CMOCs were synthesised into a final programme theory operating at five levels to influence implementation feasibility, fidelity, and sustainability: (1) the bCPAP device, (2) local partnerships and infrastructure, (3) clinical and technical teams, (4) caregivers and the community, and (5) institutional practices.

**Interpretation:**

Using realist methods with a diverse, international stakeholder panel, we generated a theory that could explain how bCPAP therapy works in different contexts. This theory could be leveraged to enhance the rigour of future bCPAP implementation trials.

**Funding:**

Yale National Clinician Scholars Program, US National Center for Advancing Translational Science (TL1TR001864), and National Heart, Lung, and Blood Institute (T32HL155000).

## Introduction

Lower respiratory tract infections (LRTIs) are the leading cause of death among children aged 1 to 59 months globally, resulting in an estimated 537 000 child deaths in 2021.^1^ Most LRTI-related child deaths occur in low-income and middle-income countries (LMICs),^2^ where health facilities often have insufficient human and physical capacity to provide uninterrupted oxygen therapy, continuous patient monitoring, invasive mechanical ventilation, or direct medical doctor supervision of care for children with LRTI.^3,4^ It is vital to implement cost-effective, evidence-based interventions to reduce mortality in these settings.

Bubble continuous positive airway pressure (bCPAP) is a promising, low-cost technology for treating children with respiratory distress, including those with LRTIs. In settings with a medical air or oxygen source, bCPAP devices can be locally improvised from modified nasal prongs and a water reservoir for as little as US$3 or purchased commercially for $800–6000 per unit.^5^ These devices are integral components of bCPAP therapy, which involves multiple concurrent services and complex interactions among interdisciplinary team members and patients’ families to ensure the appropriate device is used in the right way for the right patient. Randomised controlled trials (RCTs) of bCPAP therapy to treat paediatric LRTI in LMICs have shown mixed results, with one trial showing an 11% absolute mortality reduction compared with low-flow-oxygen,^6^ a second identifying a 4% absolute mortality reduction in infants but no significant difference in children aged 1–5 years,^7^ and a third reporting an absolute 6% increase in mortality.^8^ One possible explanation for these differing results is the wide between-trial variation in bCPAP therapy interventions, patient selection, implementation processes, and clinical settings. Indeed, systematic reviews published in 2022 concluded that the effectiveness of bCPAP for children in LMICs is context-dependent, identified factors associated with bCPAP effectiveness and safety, and highlighted the need to more rigorously examine contextual factors and mechanisms influencing bCPAP implementation in LMICs using theories of change.^9,10^

Realist review methodology is one such theory-informed approach to understanding how variations in context and mechanisms drive differences in outcomes. Realist reviews encourage the use of multiple data sources, which enables the integration of different perspectives. This approach allows authors to go beyond the question of what works to answer the more relevant question of what works for whom under which circumstances.^11–14^ Thus, we conducted a realist review to understand contextual factors influencing the safe and effective implementation of bCPAP to treat respiratory distress in children aged 1–59 months in LMICs and to develop a theory to guide safe and effective bCPAP implementation in this population.

## Methods

### Study design and initial programme theory

We conducted a realist review because realist methods are particularly suitable for understanding how complex interventions with multiple components, such as bCPAP therapy, work and under what circumstances.^15^ In our realist review, we developed an initial programme theory (IPT) explaining why bCPAP therapy might or might not be safe and effective in different contexts. We refined our IPT into context–mechanism–outcome configurations (CMOCs) by conducting a literature review, stakeholder surveys, focus groups, and key informant interviews. Furthermore, we integrated our CMOCs into a final programme theory.

We define the terms used in our realist review, adapted from methodological literature,^15–17^ in the panel. We conceptualised mechanisms as bCPAP therapy components (mechanistic resources) that change human behaviour or reasoning (mechanistic response) in a specific context, causing a specific outcome. We report this review following the RAMESES guidelines (appendix pp 2–3).^18^ We registered the protocol with PROSPERO (CRD42023403584). The Yale Institutional Review Board (protocol 2000035766) exempted the study from review, classifying it as minimal risk interview and survey research.

We developed an IPT (appendix p 4) based on a review of a purposefully diverse sample of 11 studies^6–8,19–26^ and input from nine members of the core review team with relevant clinical experience. NI and MN reviewed the 11 studies and extracted contextual factors associated with implementation outcomes relevant to safe and effective bCPAP use to treat respiratory distress in children aged 1–59 months in LMICs. Core review team members developed and validated these associations and discussed possible underlying mechanisms during a virtual meeting to generate nine candidate CMOCs (appendix p 4).

In our IPT, we hypothesised many possible factors interacting at different levels to drive bCPAP safety and effectiveness. Although we broadly considered bCPAP device type, we did not include factors internal to the bCPAP device setup (eg, the size and fit of the nasal interface or the diameter of respiratory tubing) or patient selection factors in our review. We made this decision because, although there is one study comparing bCPAP device setups in a simulated model,^27^ we were unable to find evidence relating specific device setups and bCPAP indications to patient outcomes in clinical settings.

### Search strategy and selection criteria

MCF, an experienced health science librarian, conducted a medical subject heading analysis of the purposively sampled studies. We iteratively translated and refined searches between databases. To maximise sensitivity, the formal search used controlled vocabulary terms and synonymous free-text words to capture the concepts of continuous positive airway pressure and LMIC (defined by World Bank criteria).^28^

On Dec 20, 2022, and July 25, 2023, MCF searched the following databases without date or language limits: MEDLINE ALL (Ovid), Embase (Ovid), Global Health (Ovid), Cumulative Index to Nursing and Allied Health Literature (CINAHL), and the Web of Science Core Collection. To identify grey literature, we conducted Google searches of the websites of relevant agencies, non-governmental organisations, and health-care facilities identified as bCPAP implementers in our peer-reviewed literature search (appendix pp 5–13) on July 18, 2023.

Two independent reviewers (NI and MN or NI and MP) conducted title and abstract screening and full text review of identified articles. Articles were deemed relevant if they reported on the use of any bCPAP device to treat respiratory distress in children younger than 5 years treated in hospital in an LMIC setting and described related contexts, mechanisms, or outcomes. We excluded articles reporting bCPAP use exclusively in neonates (age <1 month) and articles that were deemed irrelevant, such as articles about non-bCPAP devices.

Although we assessed the rigour of randomised trials, pre–post interventional studies, quantitative observational studies, economic evaluations, and qualitative studies using the appropriate Critical Appraisal Skills Programme (CASP) checklist, we did not exclude any studies on the basis of rigour. CASP checklists do not provide an overall appraisal score, preventing our team from inadvertently assigning more weight to articles because of a score rather than its contribution to theory development. We assessed the rigour of other peer-reviewed and grey literature by assessing trustworthiness of the source and congruence with other literature. The two independent reviewers resolved all differences in assessments of relevance and rigour through discussion based on the mentioned criteria, involving the core research team as necessary.

Three research team members (NI, MN, and MP) extracted descriptions of contexts, mechanisms, or outcomes (as identified in our IPT) from included articles using NVivo 14 (Lumivero, Denver, CO, USA). We also extracted descriptions that expanded or refuted our IPT, focusing on broader themes including bCPAP device systems and setups, bCPAP clinical protocols, infrastructure and facilities, costs and maintenance, and people and culture.

### Data analysis and theory refinement

We used two coding frameworks. In the first framework, we used both deductive logic (from our IPT and core review team discussions, such as staffing, infection control, and patient monitoring) and inductive logic (from literature analysis, such as family concerns, effective communication, and referral system) to assign codes to extracted data. NI, MN, and MP met every 1 to 2 weeks to identify each of these codes as a context, mechanism, or outcome; codes were refined as needed to meet our definitions of these terms (panel).

In the second framework, we developed a separate code for each CMOC and assigned these CMOC codes to statements from the literature linking contexts, mechanisms, and outcomes as configurations. This framework initially included CMOCs from our IPT. We refined these and generated new CMOCs by noting co-occurring contexts and outcomes, using evidence from the literature to identify possible underlying causal mechanisms. Data analysis started during data extraction; as CMOCs changed, we updated code definitions and revisited previously coded articles to ensure accurate coding in accordance with the new definitions. Each CMOC comprised at least one context, mechanism (disaggregated into resource and response), and outcome. The core review team met every two weeks to align codes and resolve differences through iterative team discussion and consensus.

During the analysis phase, we decided to focus on three proximal implementation outcomes (feasibility, fidelity, and sustainability). We based this decision on previous literature suggesting that gaps in feasibility,^23,29–33^ fidelity,^8,34^ and sustainability^31^ played important roles in undermining bCPAP safety and effectiveness in inpatient settings.

During the data analysis and theory refinement stages, we assembled a panel of stakeholders with field experience with different components of bCPAP therapy in LMICs to test the CMOCs under development and inform our final programme theory. We identified stakeholders by their authorship of randomised, controlled bCPAP trials, membership in relevant professional societies, and experience developing relevant guidelines. We identified additional stakeholders through snowball sampling, with purposive selection to ensure diverse representation by sex, discipline, and WHO region.

We iteratively solicited stakeholder input through surveys (Qualtrics, Provo, UT, USA) and virtual focus groups. Each survey presented the most recent CMOCs and asked stakeholders to indicate their level of agreement with each CMOC (ie, agree with statement as written, agree with intent, or disagree) and explain any disagreements. Stakeholders could also indicate if a particular CMOC was outside of their area of expertise.

After obtaining informed consent, we invited all stakeholders to attend each focus group and shared aggregated, anonymised survey results in advance. During each focus group, the lead author (NI) facilitated a discussion focused on the CMOCs that generated the most disagreement or comments in the preceding survey. Two participants unable to attend any focus groups participated in similarly structured interviews. The core team reviewed stakeholder input using field notes and audio recordings and met regularly between consecutive rounds of surveys and focus groups to revisit previously coded literature and revise CMOCs until thematic saturation was reached. We integrated interrelated CMOCs into a final programme theory, abstracting CMOC outcomes to the level of three proximal implementation outcomes operating sequentially: feasibility, fidelity, and sustainability.

### Role of the funding source

The study funders had no role in study design, data collection, data analysis, data interpretation, or writing of the report.

## Results

**Figure 1:**
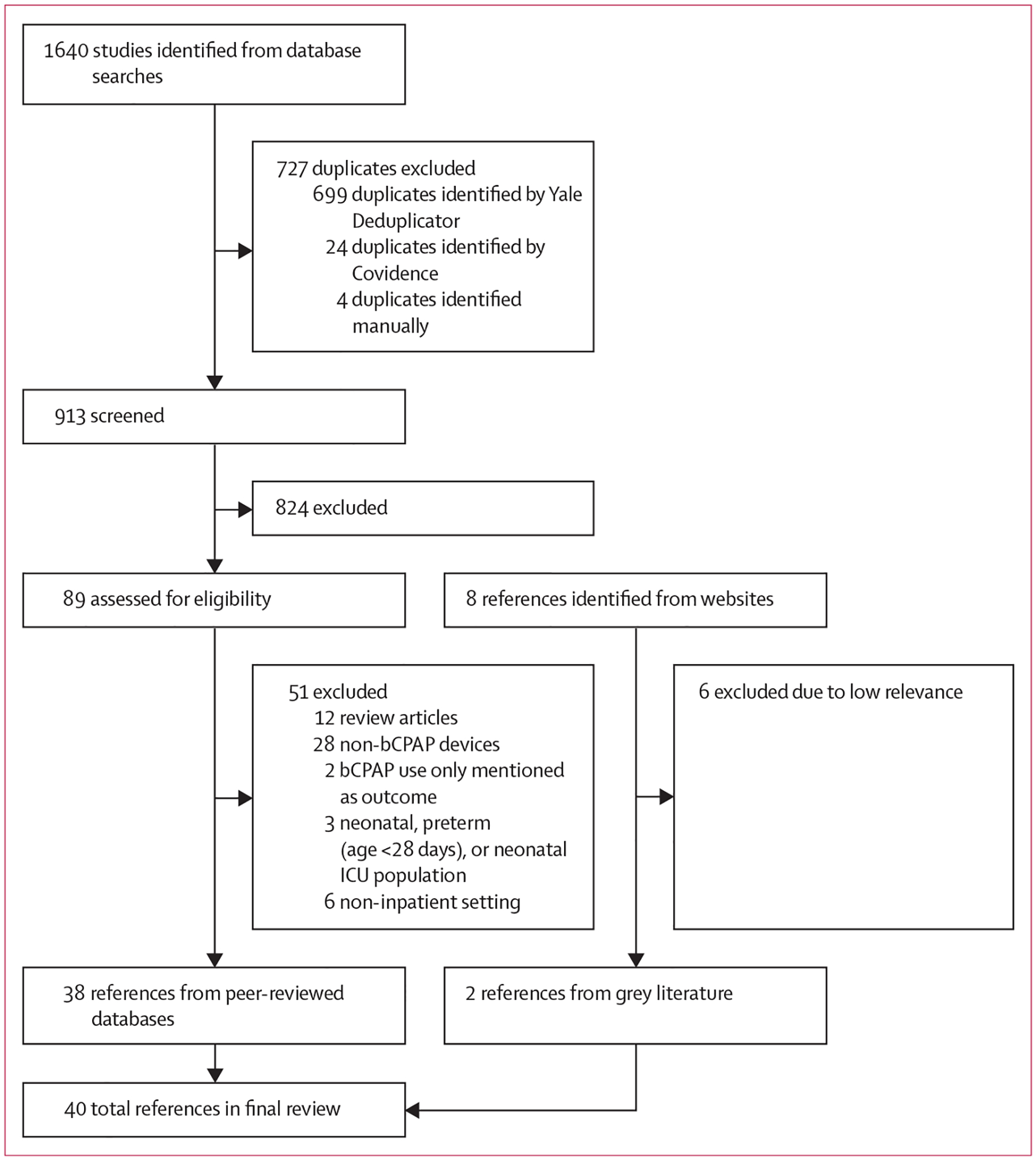
Flow diagram for selection of included articles from the peer-reviewed literature bCPAP=bubble continuous positive airway pressure. ICU=intensive care unit.

The final search retrieved a total of 1640 peer-reviewed references and eight grey literature documents. For peer-reviewed references, we removed duplicates using the Yale Deduplicator (New Haven, CT, USA) and Covidence (Melbourne, VIC, Australia) tools, leaving 913 articles for screening. Of these 913 peer-reviewed articles and eight grey literature documents, 38 peer-reviewed articles and two grey literature documents met our inclusion criteria (figure 1; appendix p 14–17). Among the 40 included articles, there were five (13%) RCTs,^6–8,19,35^ two (5%) trial protocols,^36,37^ two (5%) pre–post interventional studies,^21,38^ ten (25%) quantitative observational studies,^22,29,39–46^ one (3%) cost-effectiveness analysis,^20^ five (13%) qualitative studies,^23,24,30,34,47^ two (5%) cross-sectional surveys,^31,48^ 11 (28%) other peer-reviewed references,^25,26,32,33,49–55^ and two (5%) grey literature documents (table 1).^56,57^ We found mixed study rigour (appendix pp 18–28).

Our final programme theory comprises 18 CMOCs from our literature review that were iteratively refined via four stakeholder surveys and three focus groups from September 2023 through January 2024. Our stakeholder panel comprised 22 individuals representing multiple disciplines and all WHO regions (table 2).

We found variation in our proximal implementation outcomes of interest—bCPAP feasibility, fidelity, and sustainability—in both the literature and stakeholder experience. Multiple articles identified bCPAP as a feasible intervention based on low cost and ease of use;^6,21,40,42,50^ others highlighted bCPAP therapy components (eg, frequent bCPAP pressure titration and continuous provision of high oxygen flow) that might be infeasible in specific contexts.^23,29–33^ Fidelity also varied: for example, only 40 (13%) of 321 patients receiving bCPAP in a large trial associating bCPAP with increased mortality had nasogastric tubes placed,^8^ and a qualitative study^34^ and stakeholders in our panel described examples of parents removing bCPAP interfaces from their child’s nares, which disrupts therapy. There were also differences in sustainability: a study in Ghana reported equipment non-functionality and decreased bCPAP-related knowledge among nurses trained by more senior nurses who received bCPAP training as part of a trial,^31^ whereas the bCPAP setup used in a trial in Bangladesh has continued to be used locally for over a decade.^6^

**Figure 2:**
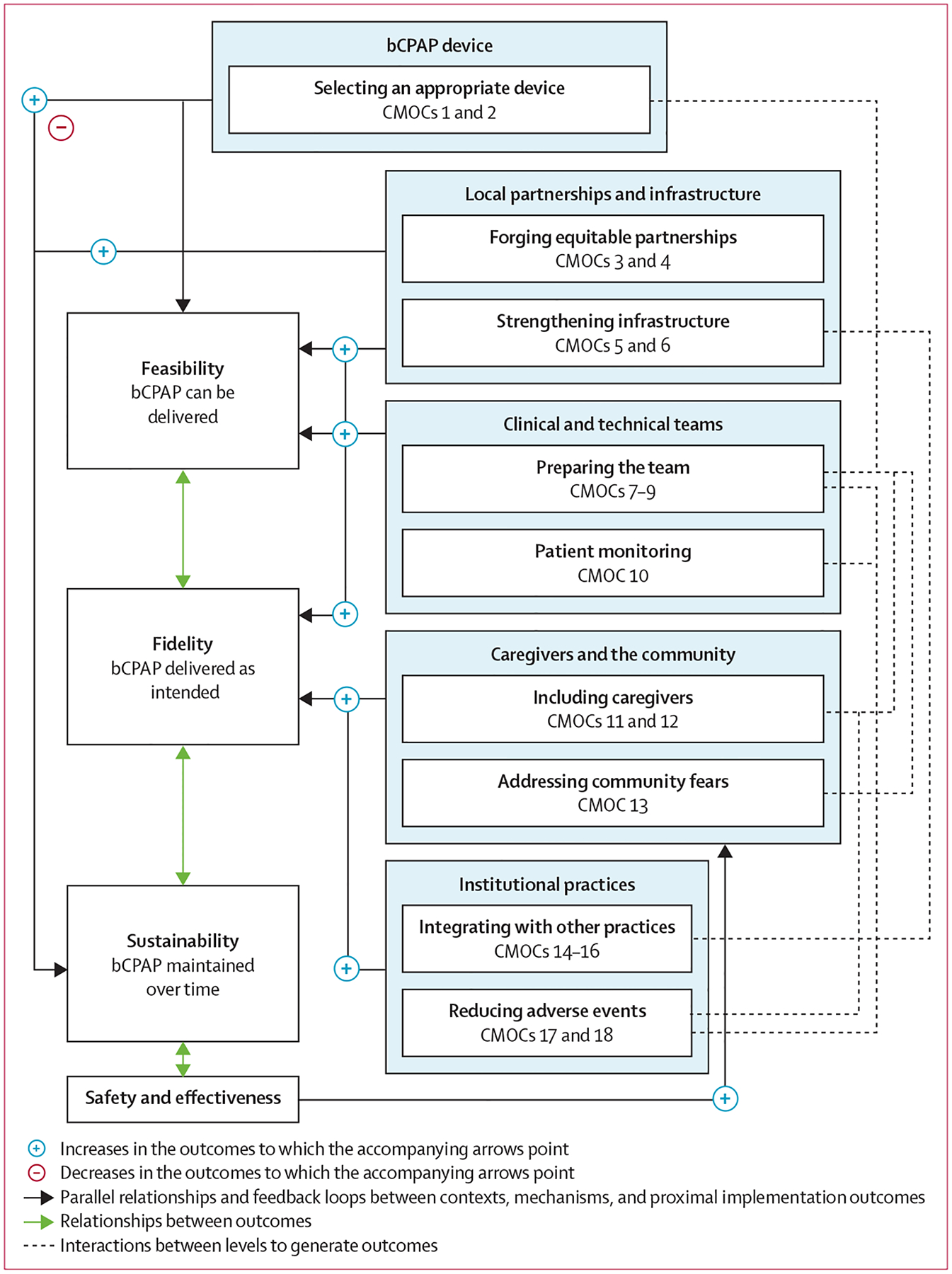
Final programme theory On the right, each box represents a level of related CMOCs that work synergistically to bring about outcomes. The levels operate sequentially. bCPAP=bubble continuous positive airway pressure. CMOC=context–mechanism–outcome configuration.

Our final programme theory acts at five sequential levels to influence bCPAP feasibility, fidelity, and sustainability: (1) bCPAP device, (2) local partnerships and infrastructure, (3) clinical and technical teams, (4) caregivers and the community, and (5) institutional practices. We depict our final programme theory in figure 2, describe the CMOCs it comprises and their supporting evidence in table 3, and provide additional evidence from the literature and stakeholders in the appendix (pp 29–58). Of note, we use the term health-care worker (HCW) to refer to anyone who works in a patient-facing clinical capacity (eg, medical doctor, nurse, and respiratory therapist), caregiver to refer to any family member accompanying a child to hospital, and bCPAP to refer to bCPAP therapy, unless noted otherwise.

Selection of an appropriate bCPAP device for a specific context is often the first step in the implementation process and heavily affects both feasibility and sustainability. Although low-cost, locally made devices that follow appropriate standards could build local expertise and resources and enhance sustainability (CMOC 1), they are often not tested and validated in clinical settings, unlike commercial bCPAP devices. Locally made bCPAP devices therefore either require local monitoring of the generated pressures and fraction of inspired oxygen or must follow the original configuration of, and use the same components as, a previously published and rigorously tested circuit without modifications. Some devices might also be easier to use, interacting with CMOC 9 to improve the confidence of HCWs in their training and ability to provide bCPAP therapy (CMOC 2).

During or after the selection of a device, local partnerships can influence feasibility and sustainability through multiple mechanisms. In settings with limited financial resources, advance budgeting with key stakeholders can facilitate the appropriate allocation of resources to bCPAP (CMOC 3). Institutional arrangements and priorities in these contexts could be influenced by external donors and researchers. In these contexts, early post-study sustainability planning with local team members can create opportunities for the longitudinal involvement of leaders with local influence (CMOC 4). Although CMOCs 3 and 4 occur early in the implementation process, they can substantially influence long-term bCPAP sustainability.

Contexts are also shaped by local infrastructure. In settings without reliable oxygen and power supply, backup sources of oxygen and electricity reduce disruptions in care, improving HCWs’ perception of bCPAP safety, which improves feasibility and fidelity (CMOC 5). Although rarely possible in contexts with limited infrastructure, in settings without intensive care capabilities, coordination with higher-level referral centres enables HCWs to seek support when necessary for patients with more severe respiratory distress needing advanced therapies, improving fidelity to clinical indications for bCPAP use (CMOC 6).

After bCPAP device selection and strengthening of local partnerships and infrastructure, bCPAP implementation depends heavily on local clinical and technical teams. However, many settings face challenges with inadequate HCW staffing. When a medical doctor is not present or available, team communication (eg, by telephone) and supportive supervision (eg, by a physically present, experienced HCW who is not a medical doctor) could enable bedside HCWs to make time-sensitive decisions and foster collaborative rather than hierarchical interdisciplinary care delivery, which improves feasibility and fidelity (CMOC 7).

Although not always possible, in settings with adequate HCW staffing, assignment of HCWs to bCPAP patients based on census and acuity allows staff to appropriately allot time for bCPAP setup, monitoring, and adjustments, which improves fidelity (CMOC 8).

CMOCs 7 and 8 require comprehensive, ongoing, and hands-on training of HCWs and technical staff. Such training, when tailored to the local context and followed by a display of competency, results in retention of knowledge and skills and emergence of local champions, particularly in settings without previous bCPAP experience, high HCW and technical staff turnover, or both, improving feasibility and fidelity (CMOC 9). In non-intensive care settings, designating a specific area for patients requiring bCPAP lowers cognitive load for HCWs by enhancing physical clustering of patients requiring similar monitoring, improving feasibility (CMOC 10). CMOC 10 feeds back to CMOCs 7 and 8 and allows targeted training of HCWs working in specific care areas (CMOC 9), synergistically enhancing feasibility and fidelity.

Once clinical teams are prepared, trained, and restructured (CMOCs 7–10), they can begin to meaningfully include caregivers in their child’s care. Where caregivers are present at the bedside, caregiver education and HCW training in responding to caregiver concerns allow caregivers to feel empowered and psychologically safe to alert HCWs of danger signs and assist in their child’s care. Caregivers alerting HCWs and HCWs responding in turn improves fidelity by reducing adverse events, especially in settings with inadequate numbers of HCWs (CMOC 11). Relatedly, explaining to caregivers the indications for and risks and benefits of bCPAP before initiation decreases caregiver distress and increases acceptance of bCPAP, decreasing the risk of caregiver-initiated disruptions in care and enhancing fidelity (CMOC 12).

In settings with high mortality rates where community members might associate oxygen therapy with death, community education and outreach reduce fears, improving timeliness of care-seeking and acceptance of bCPAP. Timeliness and acceptance enhance fidelity, because patients for whom bCPAP is intended are more likely to present to care and receive bCPAP at a time when it could be beneficial (CMOC 13). Trained teams (CMOC 9) can facilitate community education, and bCPAP safety and effectiveness further reduce community fears in a positive feedback loop (figure 2).

Consideration of how bCPAP interacts with other institutional practices also improves fidelity. In settings with previous adoption of basic respiratory care practices (eg, providing low-flow oxygen and nasopharyngeal suctioning as needed), integration of bCPAP within existing functional respiratory care services results in HCWs perceiving bCPAP as an extension of these services, improving adherence to intended protocols and fidelity (CMOC 14). In settings with experience of successfully adapting guideline-recommended practices to the local context using a quality improvement approach, evidence-based, locally relevant bCPAP clinical use guidelines normalise intended bCPAP care practices, improving fidelity (CMOC 15). CMOCs 14 and 15 depend heavily on infrastructure strengthening to reliably provide basic respiratory care services, including oxygen therapy (CMOC 5).

At times, other new initiatives might be launched concurrently with bCPAP. In these situations, a coordinated roll-out strategy (ie, combined, parallel, or stepwise roll-out) that considers whether initiatives complement or conflict with bCPAP could optimise the allocation of existing human resources to bCPAP implementation, reducing the effect on HCW cognitive load, thereby improving fidelity (CMOC 16).

Also at this level are institutional practices to reduce adverse events. Some settings have previously adopted adverse event prevention strategies (eg, gastric tubes for decompression and head of bed elevation to 30° to prevent aspiration). When these strategies are integrated with bCPAP, HCWs feel more competent and confident in using these strategies with bCPAP (eg, placing a nasogastric tube while having a nasal bCPAP interface in place), reducing adverse events, and enhancing fidelity (CMOC 17). Additionally, many settings reuse single-use consumables due to resource limitations. In these settings, following a safe and standard protocol for cleaning, disinfection, sterilisation, and cessation of reuse results in the mitigation of infection risk and recognition of material degradation (CMOC 18). CMOC 18 works synergistically with staff training, patient monitoring, and caregiver empowerment (CMOCs 9–11) to reduce adverse events and improve fidelity.

## Discussion

In this realist review with input from an interdisciplinary, global stakeholder panel, we generated a final programme theory that describes contextual factors influencing bCPAP implementation and how bCPAP therapy components might trigger different mechanisms in different contexts to reach specific outcomes for children aged 1–59 months with respiratory distress in LMICs. In doing so, this realist review directly addresses calls made by two systematic reviews published in 2022 for a theory-driven, implementation-focused approach to better elucidate why bCPAP-related outcomes might be context-dependent.^9,10^ Additionally, we expanded our understanding of the complex array of contextual and mechanistic factors influencing bCPAP implementation at five levels, including the bCPAP device, local partnerships and infrastructure, clinical and technical teams, caregivers and the community, and institutional practices.

As the bCPAP field increasingly recognises the need for implementation research, our theory and its subcomponents can directly inform the development of robust, evidence-based context, process, and outcome measures for implementation trials, tailored to implementation level. Although a previous trial included some fidelity measures,^8^ most trials have not measured implementation rigorously. In filling this gap, our theory could greatly enhance the rigour of future research.

We envision that implementation practitioners, policy makers, and funders can leverage our programme theory (figure 2) to better identify where resources are needed for bCPAP implementation. First, bCPAP implementation is complex, involves many factors aside from the device itself, and successful implementation depends on programme development and investments at multiple levels. Second, the inclusion of caregivers in caring for their children and the addressing of community fears (CMOCs 11–13), although historically overlooked, deserve attention as potential enhancers of implementation outcomes, either in synergy with other factors (ie, staff training and reducing adverse events) or as alternative pathways to attain outcomes in particular contexts. Third, CMOCs that most affect bCPAP sustainability occur early in the sequence (CMOCs 1–6). Sustainability must be considered early in the implementation process to maximise potential for success.

Relevant to HCWs, bCPAP implementation depends heavily on clinical and technical teams (CMOCs 7–10). In agreement with one review and guidelines published in 2023,^10,58^ we found that the physical presence of a bCPAP-trained medical doctor is ideal for patient safety. Using realist methods, we build on this finding by highlighting other mechanisms by which bCPAP safety can be improved in non-intensive care unit settings (CMOC 10) or settings with inadequate medical doctor presence (CMOC 7). Our theory-driven understanding of these alternative mechanisms advances the field from singular, expert opinion-driven statements (eg, “the use of continuous positive airway pressure should be conducted under physician oversight”)^58^ to an evidencedriven theory that embraces the multiple complex pathways influencing bCPAP outcomes.

Similarly, we were able to contextualise existing research that used simulated lung models to show that some low-cost and locally improvised bCPAP devices could deliver unreliable pressures compared with higher-cost, commercial devices.^27,59,60^ Although we did not include these or other simulation studies in our literature review, we did discuss this body of evidence at length during our stakeholder panel focus groups. Coupling multiple studies reporting large mortality reductions with locally improvised devices and the experiences of multiple stakeholders successfully implementing these devices in LMICs,^6,36,47,61^ we highlighted the mechanisms by which the use of locally improvised devices can improve sustainability, while acknowledging the need to follow validated device configurations and monitor quality and safety (CMOCs 1 and 2; figure 2).

Several potential limitations require consideration. First, new publications in this rapidly evolving field, most notably a randomised trial showing benefit of locally improvised bCPAP in Ethiopian general hospitals, have emerged since our literature search and stakeholder focus groups.^61^ However, because the trial’s senior author was on our stakeholder panel, we were able to incorporate the key implementation-related learnings from this trial into our CMOCs. Second, we limited the scope of our review to include only CMOCs that emerged from our literature search. Because we could not identify relevant evidence in the literature, we did not develop CMOCs related to other topics—including policy and regulation (eg, international device procurement and local improvisation standards) and medical waste associated with bCPAP use—that emerged from our stakeholder panel. Third, although we did not include caregivers of children requiring bCPAP in our stakeholder panel for practical reasons, our literature review included several qualitative studies that reported data from parents, guardians, and other caregivers.^23,24,34,47^ Fourth, we only included one study and two stakeholders from upper-middle-income countries in our review, potentially limiting the generalisability of our findings to these contexts.

This realist review offers a pragmatic theory for bCPAP implementation in LMICs. This theory could provide a mechanistic explanation for the different outcomes observed among the large bCPAP trials in LMIC contexts. Furthermore, this theory can be used to enhance the rigour of future implementation trials; provide a basis for the design of context, process, and outcome measures; tailor implementation strategies to local contexts; and inform the restructuring of clinical and technical teams to support bCPAP implementation. Our theory could also be adaptable to other emerging advanced respiratory care modalities (eg, high-flow nasal cannula oxygen) in LMICs. Future field research should focus on developing, operationalising, and testing bCPAP implementation strategies with input from local stakeholders, including HCWs, technical staff, and caregivers.

## Supplementary Material

Supplementary Appendix

## Figures and Tables

**Table 1:** Characteristics of peer-reviewed studies included in realist review, grouped by study type

	Reference type	Sample size	Setting	Study objectives	Patient population	bCPAP device type	bCPAP interface	bCPAP oxygen blending	Comparison intervention	Primary outcome
**Trials**										
Chisti et al (2015),^6^ Bangladesh	RCT	225	Tertiary care hospital ICU	Assess whether bCPAP improved outcomes compared with low-flow and high-flow oxygen	Children younger than 5 years with WHO-defined severe pneumonia and SpO_2_ <90%	Locally improvised	Standard nasal prongs	No	Nasal cannula and high-flow nasal cannula oxygen	Composite treatment failure
Gebre et al (2022),^36^ Ethiopia	Cluster RCT protocol	··	12 district hospitals	Assess bCPAP effectiveness compared with WHO standard low-flow oxygen	Children aged 1–59 months with WHO-defined severe pneumonia and SpO_2_ <90%	Locally improvised	Standard nasal prongs	No	Nasal cannula and high-flow nasal cannula oxygen	Composite treatment failure
Lal et al (2018),^19^ India	RCT	72	Tertiary care hospital	Evaluate bCPAP efficacy in decreasing respiratory distress in bronchiolitis	Infants younger than 1 year with clinical bronchiolitis	Locally improvised	Unknown	Yes	Oxygen mask	Change in respiratory rate and severity scores
McCollum et al (2019),^8^ Malawi	RCT	1712	District hospital general paediatric ward	Establish whether bCPAP reduces mortality compared with low-flow oxygen	Children aged 1–59 months with WHO-defined severe pneumonia and HIV, severe malnutrition, or SpO_2_ <90%	Commercially manufactured	CPAP nasal mask or prongs	No	Nasal cannula oxygen	Survival to hospital discharge
Smith et al (2017),^37^ Malawi	RCT protocol	··	District hospital general paediatric ward	Establish whether bCPAP reduces mortality compared with low-flow oxygen	Children aged 1–59 months with WHO-defined severe pneumonia and HIV, severe malnutrition, or SpO_2_ <90%	Commercially manufactured	CPAP nasal mask or prongs	No	Nasal cannula oxygen	In-hospital mortality
Wilson et al (2013),^35^ Ghana	RCT	70	Four district hospital emergency wards	Assess whether bCPAP decreases respiratory rate	Children aged 3 months to 5 years with tachypnoea and respiratory distress	Commercially manufactured	CPAP nasal prongs	Yes	CPAP applied 1 h after presentation	Change in respiratory rate at 1 h
Wilson et al (2017),^7^ Ghana	Cluster crossover trial	2200	Two non-tertiary hospital emergency departments	Assess whether registered nurse-initiated CPAP decreases all-cause 2-week mortality	Children aged 1 month to 5 years with tachypnoea and respiratory distress	Commercially manufactured	CPAP nasal prongs	Yes	Non-rebreather face mask oxygen	All-cause mortality at 2 weeks
**Pre–post interventional studies**										
Bjorklund et al (2019),^21^ Uganda	Pre–post interventional study	132	Paediatric acute care unit at regional referral hospital	Show safety of a new modified bCPAP device	Children aged 30 days to 5 years with moderate to severe respiratory distress or SpO_2_ <90% despite low-flow oxygen	Locally improvised	Modified nasal prongs	No	Historical control before bCPAP implementation	Adverse event composite variable
Olayo et al (2019),^38^ Kenya	Pre–post interventional study	37 HCWs	Ten different referral hospitals	Assess whether training-of-trainers curriculum can decrease gaps in skills and knowledge between first-generation and second-generation HCWs	Kenyan medical doctors, medical officers, clinical officers, and registered nurses	Commercially manufactured	CPAP nasal prongs	Yes	NA	Skills and knowledge test scores
**Quantitative, observational studies**										
Browde and Morrow (2019),^22^ South Africa	Prospective cohort study	31	Tertiary care hospital high-care ward unit and paediatric ICU	Describe characteristics and outcomes of children who received nasal CPAP	Children aged 0 to 12 years without primary lung pathology who received nasal CPAP or high-flow nasal cannula	Commercially manufactured	CPAP nasal prongs	Yes	NA	NIV failure rate
Buys et al (2023),^39^ South Africa	Retrospective cohort study	500	Tertiary care hospital emergency unit	Describe cohort receiving bCPAP	All children on bCPAP	Commercially manufactured	CPAP nasal prongs	Yes	NA	bCPAP ventilation failure rate
Jayashree et al (2016),^40^ India	Prospective cohort study	330	Teaching and referral hospital	Evaluate utility of bCPAP in hypoxaemic clinical pneumonia	Children aged 1 month to 12 years with pneumonia and respiratory distress or SpO_2_ <92%	Locally improvised	Standard nasal prongs	No	NA	Proportion of patients requiring intubation
Kinikar et al (2011),^41^ India	Prospective cohort study	36	Tertiary care hospital	Assess safety and effectiveness of indigenously assembled bCPAP during swine flu pandemic	Children aged 0 to 12 years with influenza-like illness and respiratory failure (FiO^2^ requirement >40% to maintain SpO_2_ >94%)	Locally improvised	Standard nasal prongs	No	NA	Physiological change before and after 6 h on bCPAP
Machen et al (2015),^42^ Malawi	Prospective cohort study	79	Tertiary care hospital acute care paediatric unit	Describe outcomes of patients treated with novel, low-cost, standalone bCPAP system	Patients who weighed ≤10 kg with respiratory distress of any cause if on bCPAP	Commercially manufactured	CPAP nasal prongs	Yes	NA	Change in respiratory severity score by 24 h
Myers et al (2019),^43^ Malawi	Prospective cohort study	117	Tertiary care hospital	Evaluate role of bCPAP in routine care of children who were critically ill	Children aged 0–48 months with severe respiratory distress in emergency zone or high-dependency unit	Locally improvised	CPAP nasal prongs	Yes or no	NA	Survival to hospital discharge
Pulsan et al (2019),^44^ Papua New Guinea	Prospective cohort study	64	Teaching hospital children’s ward intensive care area and special care nursery	Evaluate the use of bCPAP in a specific patient population	Children with severe pneumonia with SpO_2_ <90% or severe respiratory distress despite standard oxygen therapy	Locally improvised	Low-resistance nasal prongs	Yes	NA	Survival to hospital discharge
Punn et al (2022),^45^ India	Prospective cohort study	115	Teaching hospital paediatric ICU	Assess clinical profile and outcome of children requiring NIV	All children aged 1 month to 18 years with respiratory distress or failure admitted to paediatric ICU on NIV	Locally improvised	Standard nasal prongs	Yes	NA	Physiological change with NIV at 2 h; NIV failure
Sessions et al (2019),^29^ Malawi	Prospective cohort study	40	District hospital paediatric ward	Compare time spent by HCWs administering bCPAP and low-flow oxygen	Children aged 1 to 59 months with WHO-defined severe pneumonia and HIV, severe malnutrition, or SpO_2_ <90%	Commercially manufactured	CPAP nasal mask or prongs	No	Nasal cannula oxygen	Mean time spent by HCWs per patient at the bedside
Walk et al (2016),^46^ Malawi	Prospective cohort study	77	Referral hospital paediatric HDU and emergency ward	Examine feasibility and outcomes of ventilation assisted with bCPAP	Children aged 1 week to 14 years with progressive acute respiratory failure despite oxygen and antimicrobial therapy	Locally improvised	CPAP nasal prongs	No	NA	Survival to hospital discharge
**Economic evaluations**										
Kortz et al (2017),^20^ Malawi	Cost-effectiveness analysis	··	District and central hospitals	Assess bCPAP cost-effectiveness	Children aged 1 month to 5 years with severe, WHO-defined pneumonia	Locally improvised	Standard nasal prongs	No	Standard of care	Costs, clinical outcomes, and averted disability-adjusted life years
Chisti et al (2023),^47^ Bangladesh	Qualitative (SSIs and FGDs)	23 registered nurses, seven medical doctors, and 14 patients	Two district hospitals	Investigate feasibility of introducing bCPAP	Caregivers or parents of paediatric patients; HCWs involved in a bCPAP study	NA	NA	NA	NA	NA
Gebre et al (2022),^23^ Ethiopia	Qualitative (SSIs, hospital observations)	30 HCWs and 15 parents	Two tertiary and two general hospitals	Examine bCPAP feasibility and acceptability	Caregivers or parents of children with severe pneumonia and hypoxaemia receiving bCPAP; medical doctors and registered nurses involved in bCPAP study	NA	NA	NA	NA	NA
Gondwe et al (2017),^24^ Malawi	Qualitative (SSIs)	12 caregivers	Tertiary care hospital	Explore experiences of caregivers	Caregivers of infants aged 0 to 6 months who had received bCPAP	NA	NA	NA	NA	NA
Nyondo-Mipando et al (2020),^30^ Malawi	Qualitative (SSIs)	46 HCWs	Three district hospitals and a tertiary hospital	Explore factors that influence bCPAP implementation among HCWs	HCWs involved in care delivery and decision-making for newborn care	NA	NA	NA	NA	NA
Sessions et al (2020),^34^ Malawi	Qualitative (FGDs)	54 mothers in eight FGDs	District hospital	Assess acceptability of bCPAP and low-flow oxygen among mothers	Mothers of children aged 1 to 59 months with severe pneumonia and HIV, malnutrition, or hypoxaemia enrolled in concurrent bCPAP trial	NA	NA	NA	NA	NA
**Cross-sectional surveys**										
von Saint André-von Arnim et al (2017)^48^	Cross-sectional survey	295	Online, global survey	Assess respiratory support capabilities for children with respiratory failure in different settings	HCWs with experience managing children with acute respiratory failure	NA	NA	NA	NA	NA
Wilson et al (2014),^31^ Ghana	Skills and knowledge assessment and cross-sectional survey	28 registered nurses	Four district hospital emergency wards	Evaluate the extent to which the skills and equipment necessary for bCPAP use have been maintained 16 months after a bCPAP trial	Registered nurses trained in bCPAP use by USA researchers or local personnel	Commercially manufactured	CPAP nasal prongs	Yes	NA	Skills and knowledge assessment scores

Only peer-reviewed studies were included in the table. Other included reference types included in the review were educational assessments (Wilson et al [2017]^49^); case reports (Larsen and Poulsen [2021],^32^ McCollum et al [2011],^50^ and Morris and Wilson [2014]^26^); editorials and commentaries (Brown and De Luca [2020],^51^ Farré et al [2019],^52^ McCollum et al [2017],^53^ Wilson [2019],^33^ and Baiden and Wilson [2021]^25^); reviews (Duke [2014],^54^ Gulla et al [2021],^55^ and PATH [2021]^56^); and a manual (WHO [2016]^57^). Sample sizes are only indicated for study types for which this is relevant. Unless otherwise indicated, sample size refers to the number of patients enrolled in the study. bCPAP=bubble CPAP. CPAP=continuous positive airway pressure. FiO_2_=fraction of inspired oxygen. FGD=focus group discussion. HCW=health-care worker. HDU=high-dependency unit. ICU=intensive care unit. IDI=in-depth interview. NA=not applicable. NIV=non-invasive ventilation. SpO_2_=oxygen saturation. SSI=semi-structured interview.

**Table 2:** Characteristics of stakeholder panel (n=22)

	Number of panel members
**Sex**	
Male	14 (64%)
Female	8 (36%)
**Professional role**	
Medical doctor	9 (41%)
Nurse	4 (18%)
Biomedical engineer	3 (14%)
Physical therapist	2 (9%)
Respiratory therapist	2 (9%)
Critical care technologist	1 (5%)
Entrepreneur	1 (5%)
**WHO region**	
Africa*	8 (36%)
Americas†	6 (27%)
Southeast Asia‡	3 (14%)
Europe§	2 (9%)
Eastern Mediterranean¶	1 (5%)
Western Pacific||	2 (9%)

* Represented countries were Cameroon, Ghana, Malawi, and South Africa.

† Represented countries were Brazil, Chile, USA, and Uruguay.

‡ Represented countries were Bangladesh and India.

§ Represented countries were Germany and Switzerland.

¶ Represented country was Pakistan.

|| Represented country was Singapore.

**Table 3:** Final CMOCs, with mechanisms disaggregated into resources and responses

	CMOC	Representative quote or quotes from stakeholders, the literature, or both	Supporting references
**Level 1: bCPAP device**			
1	In settings with limited financial resources (context), low-cost, locally made bCPAP devices that follow appropriate standards (resource) build local expertise and resources (response), improving sustainability (outcome)	“I have seen many of these commercially made devices being thrown here and there because the parts are not available. […] The locally improvised (devices) to me outlast the commercially available ones […] because […] you have the parts.” (medical doctor, Ghana, focus group 2)	6,20,41,47,54,55,57
2	In settings with limited staffing (context), bCPAP devices and interfaces that are easy to use (resource) facilitate HCWs feeling confident in their training and ability (response), improving feasibility (outcome)	“In response to open-ended questions, 16 of 28 [nurses] expressed a desire for more robust training. [….] Eight nurses complained about the cumbersome nature of the CPAP set-up […].” (Wilson et al [2014])^31^	6,21,23,31,32,40,42,50,54
**Level 2: local partnerships and infrastructure**			
Forging equitable partnerships			
3	In settings with limited resources (context), advance budgeting for implementation costs (resource) results in appropriate allocation of resources to bCPAP (response), improving feasibility and sustainability (outcome) of newly developed programmes	“[The budget should] include other indirect costs like training, developing local clinical guidelines, implementing biomedical engineering workshops […].” (biomedical engineer, Switzerland, survey 3)	30,34,43,46,47
4	In settings where bCPAP is introduced as a collaboration with external stakeholders (context), early post-study sustainability planning with local team members (resource) creates opportunities for the longitudinal involvement of leaders with local influence (response), leading to improved equity and sustainability (outcome)	“I don’t necessarily agree that planning for sustainability alone results in leadership/empowerment. There are steps in between. It is more likely that inclusion of local leadership/stakeholder buy-in in the early planning phase leads to empowerment and program sustainability.” (nurse, Malawi, survey 2)	25,26,52,56
Strengthening infrastructure			
5	In settings with unreliable oxygen and power supply (context), ensuring reliable backup sources of oxygen and electricity (resource) reduces disruptions in care and enhances HCWs’ perception of bCPAP safety (response), improving feasibility and fidelity (outcome)	“I would go further in the formulation: without reliable electricity systems and/or oxygen back-up options (eg, O2-cylinders) bCPAP cannot be used safely.” (medical doctor, multiple countries in sub-Saharan Africa*, survey 1)	6,23,25,30,37,39,43,47,49,53,54,56
6	In settings without ICU capabilities (context), creating a system of safe referral to a higher level of care, where available, for patients on bCPAP (resource) allows HCWs to seek external support when necessary for patients with more severe respiratory distress (resource), improving fidelity to clinical indications for bCPAP use (outcome)	“In practice I observe clinically very severe children, for whom [even] bCPAP would not be the ideal support […] to await external assistance” (physical therapist, Brazil, survey 1)	25,36,39,44,47
**Level 3: clinical and technical teams**			
Preparing the team			
7	In settings with limited medical doctor presence (context), team communication and supportive supervision (resource) enable bedside HCWs to make time-sensitive decisions and foster collaborative rather than hierarchical interdisciplinary care delivery (response), improving feasibility and fidelity (outcome)	“To successfully treat these patients, it needs more [than protocols]. And it needs […] the training and empowering to have good clinical reasoning. And that needs backup, supportive supervision, possibilities to ask a friend.” (medical doctor, multiple countries in sub-Saharan Africa*, focus group 1)	25,30,38,49,54,56
8	In settings with adequate staffing (context), assignment of staff to bCPAP patients based on census and acuity (resource) allows staff to appropriately allot time for bCPAP setup, monitoring, and adjustments (response), leading to improved fidelity (outcome)	“There is no doubt about this that there should be adequate staff, and that’s obviously going to improve the safety [….].” (medical doctor, India, focus group 3); “Time for staff is one component of this process […].” (paediatric pulmonologist and researcher, South Africa, survey 1)	39,43,46–48,55,56
9	Comprehensive, contextualised, ongoing, and hands-on clinical and technical staff bCPAP training followed by display of competency in real-world scenarios (resource) facilitates retention of knowledge and skills and emergence of local champions (mechanism), particularly in settings without previous bCPAP experience, with high staff turnover, or both (context), resulting in improved feasibility and fidelity (outcome)	“I don’t believe formal bCPAP training alone facilitates retention of knowledge, but rather acquisition of knowledge, perhaps increased competency and confidence in the skill. In my experience, facilitation of knowledge retention occurs with repeated training, follow-up, use of the skill in the practical setting, hands-on skill check off, etc […].” (nurse, Malawi, survey 2); “Once we train someone, we also empower the person to be a trainer.” (medical doctor and researcher, Ghana, focus group 1)	23,25,26,30,31,35,38,39,43,44,47–49,54–56
Patient monitoring			
10	In non-ICU settings (context), designating a specific care area for patients requiring bCPAP (resource) lowers HCW cognitive load by enhancing physical clustering of patients requiring similar monitoring (response), improving feasibility (outcome)	“You don’t need a high tech, separate space. I see it more as clustering of similar patients […] for close monitoring, which is there. [….] It’s about monitoring and its timely […] and appropriate response.” (physical therapist, South Africa, focus group 3)	36,39,43,47,54
**Level 4: caregivers and the community**			
Including caregivers			
11	In settings where a caregiver is present at the bedside (context), caregiver education and HCW training in responding to caregiver concerns (resource) allow caregivers to feel empowered and psychologically safe to alert HCWs of danger signs and assist in their child’s care (response), reducing adverse events and improving fidelity (outcome)	“What we have done in Bangladesh and Ethiopia, we educated the mother that if the mother can see [nasal secretions], mother can ask the nurses, even if the nurse might not be available at that time, and it worked dramatically. Initially, they were hesitant, but eventually they responded very well.” (medical doctor, Bangladesh, focus group 1)	30,34,43,46,47
12	In settings where a caregiver is present at the bedside (context), explanation to caregivers regarding bCPAP before therapy initiation (resource) decreases caregiver distress and increases caregiver acceptance of bCPAP (response), decreasing caregiver-initiated disruptions in care and enhancing fidelity (outcome)	“Mothers often reported being told to remove the equipment temporarily or permanently by other caregivers and this led to several self-reports of interrupting care.” (Sessions et al [2020])^34^; “In this study, the caregivers who found their infants already commenced on bCPAP were more stressed than their counterparts.” (Gondwe et al [2017])^24^	23,24,30,34
Addressing community fears			
13	In high-mortality settings where community members might associate oxygen therapy with death (context), community education and outreach (resource) reduce community fears related to inpatient care and oxygen therapy (response), improving timeliness of care-seeking, community acceptance of bCPAP, and fidelity (outcome)	“Perceptions about bCPAP treatment varied, but common to most participants was the fear of injury to or death of their child when bCPAP was initiated.” (Gondwe et al [2017])^24^	24,26,30,34,57
**Level 5: institutional practices**			
Integrating with other practices			
14	In settings with prior adoption of basic respiratory care practices (context), integration of bCPAP within existing functional respiratory care services (resource) results in HCWs perceiving bCPAP as an extension of existing care services (response), improving adherence to intended protocols and fidelity (outcome)	“Yes oxygen systems provide a framework, but CPAP is unique enough that the resource utilization is much different.” (medical doctor, South Africa, survey 1)	20,23,26,47,48
15	In settings with experience in successfully adapting guideline-recommended practices to the local context using a quality improvement approach (context), evidence-based, locally relevant bCPAP clinical use guidelines (resource) normalise intended bCPAP care practices (response), improving fidelity (outcome)	“To have global impact, it is not sufficient to simply develop an affordable, effective respiratory support system. In addition, staff must be trained to deliver the therapy, clinical guidelines and monitoring for use must be in place.” (von Saint André-von Arnim et al [2017])^48^	22,47,48,54
16	In settings with other new initiatives being implemented simultaneously with new bCPAP implementation (context), a coordinated strategy for rolling out multiple programmes (resource) results in more efficient resource allocation and reduces the effect on HCW cognitive load (response), improving fidelity (outcome)	“My thinking is that multiple initiatives implemented at the same time could be distracting for clinicians and actually make resource allocation less efficient. But I could also see how appropriately paired initiatives could have a synergistic effect.” (Nurse, Malawi, Survey 2)	29
Reducing adverse events			
17	Previous adoption of clinical adverse event prevention strategies (context), when integrated with a bCPAP programme (resource), enhance HCW competence and confidence in using these strategies with bCPAP (response), reducing bCPAP-related adverse events and enhancing fidelity (outcome)	“There can be so much trouble associated with gastric tubes that it’s a real important element of the training, especially after initial stabilization.” (medical doctor, multiple countries in sub-Saharan Africa*, focus group 1)	6,8,32,33,37,43,51,54,55
18	In settings in which single-use consumables are reused (context), following a safe and standard protocol for cleaning, disinfection, sterilisation, and cessation of reuse (resource) results in staff mitigating infection risk and recognising material degradation (response), reducing adverse events and enhancing fidelity (outcome)	“We have the three steps: […] cleaning, disinfection, and then sterilization, depending on the type of consumable. [….] Even if the process or protocol is followed well, there is a lifespan for each of the consumables, so that needs to be considered.” (biomedical engineer, Switzerland, focus group 3)	8,21,26,31,33,54

Supporting references and representative quotes from the literature and stakeholder panel are provided. bCPAP=bubble continuous positive airway pressure. CMOC=context–mechanism–outcome configuration. HCW=health-care worker. ICU=intensive care unit.

* Represented countries included Cameroon, Ghana, Malawi, and South Africa.

## Data Availability

Researchers wishing to undertake additional analyses of the data are invited to contact the corresponding author. The study protocol and analysis plan are available on PROSPERO (https://www.crd.york.ac.uk/prospero/display_record.php?RecordID=403584).

## Research in context

### Evidence before this study

Bubble continuous positive airway pressure (bCPAP) has been shown to reduce mortality in children with lower respiratory tract infections and other causes of respiratory distress in multiple trials; however, one large RCT showed an increased risk of mortality with bCPAP in children with severe pneumonia. Differences in implementation context could contribute to these different outcomes. On Dec 20, 2022, and July 25, 2023, we searched the following databases without date or language limits: MEDLINE ALL (Ovid), Embase (Ovid), Global Health (Ovid), Cumulative Index to Nursing and Allied Health Literature (CINAHL), and the Web of Science Core Collection. We used controlled vocabulary terms and synonymous free-text words (appendix pp 5–8) to capture the concepts of “continuous positive airway pressure” and “LMIC” (defined by World Bank criteria). We found 11 reviews on bCPAP use for children in low-income and middle-income countries (LMICs). We did not find any realist reviews. Two systematic reviews published in 2022 described how bCPAP implementation context might influence outcomes. In one of these papers, Sessions and colleagues reviewed 20 studies, focusing on the efficacy and safety of continuous positive airway pressure, including bCPAP, in children with respiratory distress. In the second paper, Wilkes and colleagues conducted a review of 23 studies, focusing on contextual factors influencing CPAP outcomes for children with severe pneumonia. Both reviews found mixed study quality and substantial study heterogeneity and concluded that bCPAP-related outcomes appear to be context-dependent. Both reviews highlighted the need for rigorous, theory-driven studies examining the contextual factors and mechanisms influencing bCPAP outcomes, but we found no studies in our search attempting to do so.

### Added value of this study

bCPAP therapy is a complex intervention that consists of not only a bCPAP device, but the appropriate use of this device for the right patient, involving multiple intervention components interacting at different levels. Following a realist theory-driven approach, we used a variety of data sources, including peer-reviewed literature, grey literature, and stakeholder input, to generate and refine a programme theory that may explain how and under what circumstances bCPAP therapy can be implemented safely and effectively in LMICs. We identified 18 contextual factors and proposed mechanisms by which they might influence bCPAP outcomes and integrated these into a final programme theory operating at five levels: (1) the bCPAP device, (2) local partnerships and infrastructure, (3) clinical and technical teams, (4) caregivers and the community, and (5) institutional practices.

### Implications of all the available evidence

bCPAP outcomes for children aged 1–59 months in LMICs appear to be context-dependent. Based on a synthesis of evidence from multiple sources, we have now theorised specific contextual factors and associated mechanisms by which these factors might influence bCPAP outcomes. Future research should focus on adapting bCPAP interventions and designing, operationalising, and testing implementation strategies in different contexts to empirically confirm our findings.

## *Panel*: Realist review definitions

### Context

Any condition that existed before bubble continuous positive airway pressure (bCPAP) therapy implementation

### Mechanism

How bCPAP therapy intervention components cause an outcome

### Resource

Any component of a bCPAP therapy intervention introduced in a context

### Response

Changes in human reasoning or behaviour that result in an outcome

### Implementation feasibility

The extent to which bCPAP therapy can be successfully delivered in a given setting

### Implementation fidelity

The degree to which bCPAP therapy was delivered as intended

### Implementation sustainability

The extent to which bCPAP therapy is maintained or institutionalised within a service setting over time

### Initial programme theory

The baseline conception of what bCPAP therapy is expected to do and how it is expected to work; used to guide the search strategy and data extraction

### Final programme theory

The refined conception of what bCPAP therapy is expected to do and how it is expected to work
