# Supplementary material for: Contextual factors influencing bubble continuous positive airway pressure implementation for paediatric respiratory distress in low-income and middle-income countries: a realist review
Source: Lancet Glob Health. Author manuscript; Available in PMC 2025 Feb 2. (PMC11779698; doi:10.1016/S2214-109X(24)00453-4)
Supplement: Supplementary Appendix [file NIHMS2043323-supplement-Supplementary_Appendix.pdf]

THE LANCET
Global Health

Supplementary appendix

This appendix formed part of the original submission and has been peer reviewed. We post it as supplied by the authors.

Supplement to: Ijaz N, Nader M, Ponticiello M, et al. Contextual factors influencing bubble continuous positive airway pressure implementation for paediatric respiratory distress in low-income and middle-income countries: a realist review. *Lancet Glob Health* 2024; published online Dec 12. https://doi.org/10.1016/S2214-109X(24)00453-4.

Supplementary materials: Contextual factors influencing bubble continuous positive airway pressure (bCPAP) implementation for pediatric respiratory distress in low- and middle-income countries: A realist review

Appendix 1. RAMESES guidelines. 2
Appendix 2. Initial program theory. 4
Appendix 3. Search strategy. 5
Appendix 3.1 Search strategy for peer-reviewed databases. 5
Appendix 3.2 Search strategy for grey literature. 8
Appendix 4. Reasons for exclusion at full-text stage. 14
Appendix 5. Assessments of rigor for included studies, by study type. 18
Appendix 5.1 Appraisals of randomized, controlled trials and protocols (n=7). 18
Appendix 5.2 Appraisals of pre-post interventional trials and quantitative observational studies (n=12). 19
Appendix 5.3 Appraisal of economic evaluation (n=1). 27
Appendix 5.4 Appraisals of qualitative studies (n=5). 28
Appendix 6. Final context-mechanism-outcome configurations with supporting and refuting evidence. 29

Appendix 1. RAMESES guidelines.

TITLE			Location where item is reported
1		In the title, identify the document as a realist synthesis or review	Page 1
ABSTRACT			
2		While acknowledging publication requirements and house style, abstracts should ideally contain brief details of: the study's background, review question or objectives; search strategy; methods of selection, appraisal, analysis and synthesis of sources; main results; and implications for practice.	Pages 4-5
INTRODUCTION			
3	Rationale for review	Explain why the review is needed and what it is likely to contribute to existing understanding of the topic area.	Pages 6-7
4	Objectives and focus of review	State the objective(s) of the review and/or the review question(s). Define and provide a rationale for the focus of the review.	Page 7
METHODS			
5	Changes in the review process	Any changes made to the review process that was initially planned should be briefly described and justified.	NA
6	Rationale for using realist synthesis	Explain why realist synthesis was considered the most appropriate method to use.	Page 7
7	Scoping the literature	Describe and justify the initial process of exploratory scoping of the literature.	Pages 8-9
8	Searching processes	While considering specific requirements of the journal or other publication outlet, state and provide a rationale for how the iterative searching was done. Provide details on all the sources accessed for information in the review. Where searching in electronic databases has taken place, the details should include, for example, name of database, search terms, dates of coverage and date last searched. If individuals familiar with the relevant literature and/or topic area were contacted, indicate how they were identified and selected.	Pages 9
9	Selection and appraisal of documents	Explain how judgements were made about including and excluding data from documents, and justify these.	Page 9-10
10	Data extraction	Describe and explain which data or information were extracted from the included documents and justify this selection.	Page 10
11	Analysis and synthesis processes	Describe the analysis and synthesis processes in detail. This section should include information on the constructs analyzed and describe the analytic process.	Page 10-11
RESULTS			
12	Document flow diagram	Provide details on the number of documents assessed or eligibility and included in the review with reasons for exclusion at each stage as well as an indication of their source of origin (for example, from searching databases, reference lists and so on). You may consider using the example templates (which are likely to need modification to suit the data) that are provided.	Figure 1
13	Document characteristics	Provide information on the characteristics of the documents included in the review.	Page 13, Table 1
14	Main findings	Present the key findings with a specific focus on theory building and testing.	Pages 13-19
DISCUSSION			
15	Summary of findings	Summarize the main findings, taking into account the review's objective(s), research question(s), focus and intended audience(s).	Page 19

16	Strengths, limitations, and future directions	Discuss both the strengths of the review and its limitations. These should include (but need not be restricted to) (a) consideration of all the steps in the review process and (b) comment on the overall strength of evidence supporting the explanatory insights which emerged. The limitations identified may point to areas where further work is needed.	Pages 19-22
17	Comparison with existing literature	Where applicable, compare and contrast the review's findings with the existing literature (for example, other reviews) on the same topic.	Pages 19-22
18	Conclusion and recommendations	List the main implications of the findings and place these in the context of other relevant literature. If appropriate, offer recommendations for policy and practice.	Pages 19-22
19	Funding	Provide details of funding source (if any) for the review, the role played by the funder (if any) and any conflicts of interests of the reviewers.	Pages 5 & 13

Appendix 2. Initial program theory.

Our initial program theory (IPT) consisted of the following candidate context, mechanism, and outcome relationships (dyads or triads). To avoid excluding potentially important relationships, we did not limit our IPT to specific types of outcomes.

Context	Mechanism	Outcome
Understaffed settings where staff cannot make frequent adjustments to the bCPAP system or settings	Lack of timely staff response to safety events	More complications
Nasogastric decompression cannot be safely and reliably performed		Gastric insufflation and aspiration events
Limited financial resources	Low-cost, locally-made or adapted bCPAP devices	More sustainable use
Inadequate resources for single-use consumables	A standard protocol for supply cleaning and reuse	Fewer device-associated infections
Understaffed, high-volume settings	Nursing staff experienced with bCPAP use	Safer use and fewer complications
Settings newly implementing bCPAP	Formal, regular staff bCPAP training	Better retention of relevant knowledge and skills
Limited resources	Backup oxygen source	Adverse event prevention
Understaffed, non-ICU settings	Less frequent vital signs measurement and bubble checks	More complications
	Caregivers participating in caring for their child	Family acceptance of bCPAP use

Appendix 3. Search strategy.

Appendix 3.1 Search strategy for peer-reviewed databases.

Ovid MEDLINE(R) ALL <1946 to July 24, 2023>

1	exp Child/ or Infant/ or pediatrics/ or PICU.ti. or (infant* or baby or babies or toddler* or pediatric* or paediatric*).mp.	3072115
2	Continuous Positive Airway Pressure/ or ("continuous positive airway pressure" or bcpap or cpap or nippv or "non-invasive positive pressure ventilation").mp.	18769
3	exp Developing Countries/	81049
4	((lami or lic or lics) adj3 countr*).tw.	411
5	(lmic or lmics).tw.	9791
6	(("third world" or "less developed" or developing or "under developed" or underdeveloped or "middle income" or "low* income" or "less* developed" or "under served" or underserved or poor* or deprived) adj3 (countr* or world or population* or nation* or gross domestic or gross national or gdp or gmp or economics or economy or economies)).mp.	213160
7	(afghan* or Benin* or Burkin* or Burundi* or "Central African Republic*" or Chad or chadian or Comoros or Comorian or Congo* or Eritrea* or Ethiopia* or Gambia* or Guinea* or Haiti* or "North Korea*" or "Democratic People's Republic of Korea" or Liberia* or Madagascar or Malagasy* or Malawi* or Mali* or Mozambiqu* or Nepal* or Niger* or Rwanda* or Senegal* or "Sierra Leon*" or Somalia* or "South Sudan*" or Tanzania* or Togo* or Uganda* or Zimbabw*).mp.	1138383
8	(Angola* or Armenia* or Bangladesh* or Bhutan* or Bolivia* or "Cabo Verd*" or Cambodia* or Cameroon* or Congo* or "Cote d'Ivoir*" or Djibout* or Egypt* or "El Salvador*" or Georgia* or Ghana* or Guatemala* or Hondura* or India* or Indonesia* or Jordan* or Kenya* or Kiribati or Kosov* or Kyrgyz* or Laos or laotian or "lao pdr" or Lesoth* or Mauritania* or Micronesia* or Moldov* or Mongolia* or Morocc* or Myanmar* or Burmese or Nicaragua* or Nigeria* or Pakistan* or Papua* or Philippin* or "Sao Tome*" or "Solomon Island*" or "Sri Lank*" or Sudan* or Swazi* or ESWATINI or Syria* or Tajik* or Timor* or Tunisia* or Ukrain* or Uzbek* or Vanuatu* or Vietnam* or "West Bank" or Gaza or palestina* or Yemen* or Zambia*).mp.	707629
9	(Albania* or Algeria* or Samoa* or Argentina* or Azerbaijan* or Belarus* or Belize or Bosnia* or Herzegovina* or Botswana* or Brazil* or Bulgaria* or Chinese or China or Colombia* or "Costa Rica*" or Croatia* or Cuba* or Dominica* or Guinea* or Ecuador* or Fiji* or Gabon* or Grenada* or Guyana* or Iran* or Iraq* or Jamaica* or Kazakh* or Lebanon or lebanese or Libya* or Macedonia* or Malaysia* or Maldiv* or "Marshall Island*" or Mauriti* or Mexic* or Montenegr* or Namibia* or Nauru* or Panama* or Paraguay* or Peru* or Romania* or Russia* or Samoa* or Serbia* or "South Africa*" or Lucia* or "St Vincent" or Grenadin* or Suriname* or Thailand or thai or Tonga* or Turkey or TURKIYE or turkish or Turkmen* or Tuvalu* or Venezuela* or PALAU or Belau or pelew).mp.	1594831
10	or/3-9	3163608
11	1 and 2 and 10	377

Embase (Ovid) <1974 to 2023 July 20>

1	exp infant/ or child/ or preschool child/ or toddler/ or PICU.ti. or (infant* or baby or babies or toddler* or pediatric* or paediatric*).mp.	3385651
2	bubble continuous positive airway pressure/ or ("continuous positive airway pressure" or bcpap or cpap or nippv or "non-invasive positive pressure ventilation").mp.	32770
3	developing country/ or low income country/ or middle income country/	124512
4	((lami or lic or lics) adj3 countr*).tw.	556
5	(lmic or lmics).tw.	12115
6	(("third world" or "less developed" or developing or "under developed" or underdeveloped or "middle income" or "low* income" or "less* developed" or "under served" or underserved or poor* or deprived) adj3 (countr* or world or population* or nation* or gross domestic or gross national or gdp or gmp or economics or economy or economies)).mp.	255754
7	(afghan* or Benin* or Burkin* or Burundi* or "Central African Republic*" or Chad or chadian or Comoros or Comorian or Congo* or Eritrea* or Ethiopia* or Gambia* or Guinea* or Haiti* or "North Korea*" or "Democratic People's Republic of Korea" or Liberia* or	1590852

	Madagascar or Malagasy* or Malawi* or Mali* or Mozambiqu* or Nepal* or Niger* or Rwanda* or Senegal* or "Sierra Leon*" or Somalia* or "South Sudan*" or Tanzania* or Togo* or Uganda* or Zimbabw*).mp.	
8	(Angola* or Armenia* or Bangladesh* or Bhutan* or Bolivia* or "Cabo Verd*" or Cambodia* or Cameroon* or Congo* or "Cote d'Ivoir*" or Djibout* or Egypt* or "El Salvador*" or Georgia* or Ghana* or Guatemala* or Hondura* or India* or Indonesia* or Jordan* or Kenya* or Kiribati or Kosov* or Kyrgyz* or Laos or laotian or "lao pdr" or Lesoth* or Mauritania* or Micronesia* or Moldov* or Mongolia* or Morocc* or Myanmar* or Burmese or Nicaragua* or Nigeria* or Pakistan* or Papua* or Philippin* or "Sao Tome*" or "Solomon Island*" or "Sri Lank*" or Sudan* or Swazi* or ESWATINI or Syria* or Tajik* or Timor* or Tunisia* or Ukrain* or Uzbek* or Vanuatu* or Vietnam* or "West Bank" or Gaza or palestina* or Yemen* or Zambia*).mp.	919018
9	(Albania* or Algeria* or Samoa* or Argentina* or Azerbaijan* or Belarus* or Belize or Bosnia* or Herzegovina* or Botswana* or Brazil* or Bulgaria* or Chinese or China or Colombia* or "Costa Rica*" or Croatia* or Cuba* or Dominica* or Guinea* or Ecuador* or Fiji* or Gabon* or Grenada* or Guyana* or Iran* or Iraq* or Jamaica* or Kazakh* or Lebanon or lebanese or Libya* or Macedonia* or Malaysia* or Maldiv* or "Marshall Island*" or Mauriti* or Mexic* or Montenegr* or Namibia* or Nauru* or Panama* or Paraguay* or Peru* or Romania* or Russia* or Samoa* or Serbia* or "South Africa*" or Lucia* or "St Vincent" or Grenadin* or Suriname* or Thailand or thai or Tonga* or Turkey or TURKIYE or turkish or Turkmen* or Tuvalu* or Venezuela* or PALAU or Belau or pelew).mp.	1965419
10	or/3-9	4176425
11	1 and 2 and 10	797
12	limit 11 to conference abstracts	176
13	11 not 12	621

Global Health (Ovid) <1910 to 2023 Week 26>

1	infants/ or preschool children/ or children/ or PICU.ti. or (infant* or baby or babies or toddler* or pediatric* or paediatric*).mp.	585959
2	("continuous positive airway pressure" or bcpap or cpap or nippv or "non-invasive positive pressure ventilation").mp.	927
3	Least Developed Countries/ or low Human Development Index countries/ or low income countries/ or lower-middle income countries/ or medium Human Development Index countries/	596600
4	((lami or lic or lics) adj3 countr*).tw.	198
5	(lmic or lmics).tw.	4702
6	(("third world" or "less developed" or developing or "under developed" or underdeveloped or "middle income" or "low* income" or "less* developed" or "under served" or underserved or poor* or deprived) adj3 (countr* or world or population* or nation* or gross domestic or gross national or gdp or gmp or economics or economy or economies)).mp.	1242823
7	(afghan* or Benin* or Burkin* or Burundi* or "Central African Republic*" or Chad or chadian or Comoros or Comorian or Congo* or Eritrea* or Ethiopia* or Gambia* or Guinea* or Haiti* or "North Korea*" or "Democratic People's Republic of Korea" or Liberia* or Madagascar or Malagasy* or Malawi* or Mali* or Mozambiqu* or Nepal* or Niger* or Rwanda* or Senegal* or "Sierra Leon*" or Somalia* or "South Sudan*" or Tanzania* or Togo* or Uganda* or Zimbabw*).mp.	289558
8	(Angola* or Armenia* or Bangladesh* or Bhutan* or Bolivia* or "Cabo Verd*" or Cambodia* or Cameroon* or Congo* or "Cote d'Ivoir*" or Djibout* or Egypt* or "El Salvador*" or Georgia* or Ghana* or Guatemala* or Hondura* or India* or Indonesia* or Jordan* or Kenya* or Kiribati or Kosov* or Kyrgyz* or Laos or laotian or "lao pdr" or Lesoth* or Mauritania* or Micronesia* or Moldov* or Mongolia* or Morocc* or Myanmar* or Burmese or Nicaragua* or Nigeria* or Pakistan* or Papua* or Philippin* or "Sao Tome*" or "Solomon Island*" or "Sri Lank*" or Sudan* or Swazi* or ESWATINI or Syria* or Tajik* or Timor* or Tunisia* or Ukrain* or Uzbek* or Vanuatu* or Vietnam* or "West Bank" or Gaza or palestina* or Yemen* or Zambia*).mp.	499930
9	(Albania* or Algeria* or Samoa* or Argentina* or Azerbaijan* or Belarus* or Belize or Bosnia* or Herzegovina* or Botswana* or Brazil* or Bulgaria* or Chinese or China or Colombia* or "Costa Rica*" or Croatia* or Cuba* or Dominica* or Guinea* or Ecuador* or Fiji* or Gabon* or Grenada* or Guyana* or Iran* or Iraq* or Jamaica* or Kazakh* or Lebanon or lebanese or Libya* or Macedonia* or Malaysia* or Maldiv* or "Marshall Island*"	882095

	or Mauriti* or Mexic* or Montenegr* or Namibia* or Nauru* or Panama* or Paraguay* or Peru* or Romania* or Russia* or Samoa* or Serbia* or "South Africa*" or Lucia* or "St Vincent" or Grenadin* or Suriname* or Thailand or thai or Tonga* or Turkey or TURKIYE or turkish or Turkmen* or Tuvalu* or Venezuela* or PALAU or Belau or pelew).mp.	
10	or/3-9	1498365
11	1 and 2 and 10	156

CINAHL Complete (EBSCOhost)

S10	(S3 OR S4 OR S6 OR S7 OR S8 OR S9) AND (S1 AND S2 AND S9)	124
S9	S3 OR S4 OR S5 OR S6 OR S7 OR S8	574,175
S8	(MH "Low and Middle Income Countries")	3,285
S7	TI ((Albania* or Algeria* or Samoa* or Argentina* or Azerbaijan* or Belarus* or Belize or Bosnia* or Herzegovina* or Botswana* or Brazil* or Bulgaria* or Chinese or China or Colombia* or "Costa Rica*" or Croatia* or Cuba* or Dominica* or Guinea* or Ecuador* or Fiji* or Gabon* or Grenada* or Guyana* or Iran* or Iraq* or Jamaica* or Kazakh* or Lebanon or lebanese or Libya* or Macedonia* or Malaysia* or Maldiv* or "Marshall Island*" or Mauriti* or Mexic* or Montenegr* or Namibia* or Nauru* or Panama* or Paraguay* or Peru* or Romania* or Russia* or Samoa* or Serbia* or "South Africa*" or Lucia* or "St Vincent" or Grenadin* or Suriname* or Thailand or thai or Tonga* or Turkey or TURKIYE or turkish or Turkmen* or Tuvalu* or Venezuela* or PALAU or Belau or pelew)) OR AB ((Albania* or Algeria* or Samoa* or Argentina* or Azerbaijan* or Belarus* or Belize or Bosnia* or Herzegovina* or Botswana* or Brazil* or Bulgaria* or Chinese or China or Colombia* or "Costa Rica*" or Croatia* or Cuba* or Dominica* or Guinea* or Ecuador* or Fiji* or Gabon* or Grenada* or Guyana* or Iran* or Iraq* or Jamaica* or Kazakh* or Lebanon or lebanese or Libya* or Macedonia* or Malaysia* or Maldiv* or "Marshall Island*" or Mauriti* or Mexic* or Montenegr* or Namibia* or Nauru* or Panama* or Paraguay* or Peru* or Romania* or Russia* or Samoa* or Serbia* or "South Africa*" or Lucia* or "St Vincent" or Grenadin* or Suriname* or Thailand or thai or Tonga* or Turkey or TURKIYE or turkish or Turkmen* or Tuvalu* or Venezuela* or PALAU or Belau or pelew))	281,915
S6	TI ((Angola* or Armenia* or Bangladesh* or Bhutan* or Bolivia* or "Cabo Verd*" or Cambodia* or Cameroon* or Congo* or "Cote d'Ivoir*" or Djibout* or Egypt* or "El Salvador*" or Georgia* or Ghana* or Guatemala* or Hondura* or India* or Indonesia* or Jordan* or Kenya* or Kiribati or Kosov* or Kyrgyz* or Laos or laotian or "lao pdr" or Lesoth* or Mauritania* or Micronesia* or Moldov* or Mongolia* or Morocc* or Myanmar* or Burmese or Nicaragua* or Nigeria* or Pakistan* or Papua* or Philippin* or "Sao Tome*" or "Solomon Island*" or "Sri Lank*" or Sudan* or Swazi* or ESWATINI or Syria* or Tajik* or Timor* or Tunisia* or Ukrain* or Uzbek* or Vanuatu* or Vietnam* or "West Bank" or Gaza or palestina* or Yemen* or Zambia*)) OR AB ((Angola* or Armenia* or Bangladesh* or Bhutan* or Bolivia* or "Cabo Verd*" or Cambodia* or Cameroon* or Congo* or "Cote d'Ivoir*" or Djibout* or Egypt* or "El Salvador*" or Georgia* or Ghana* or Guatemala* or Hondura* or India* or Indonesia* or Jordan* or Kenya* or Kiribati or Kosov* or Kyrgyz* or Laos or laotian or "lao pdr" or Lesoth* or Mauritania* or Micronesia* or Moldov* or Mongolia* or Morocc* or Myanmar* or Burmese or Nicaragua* or Nigeria* or Pakistan* or Papua* or Philippin* or "Sao Tome*" or "Solomon Island*" or "Sri Lank*" or Sudan* or Swazi* or ESWATINI or Syria* or Tajik* or Timor* or Tunisia* or Ukrain* or Uzbek* or Vanuatu* or Vietnam* or "West Bank" or Gaza or palestina* or Yemen* or Zambia*))	148,155
S5	TI ((afghan* or Benin* or Burkin* or Burundi* or "Central African Republic*" or Chad or chadian or Comoros or Comorian or Congo* or Eritrea* or Ethiopia* or Gambia* or Guinea* or Haiti* or "North Korea*" or "Democratic People's Republic of Korea" or Liberia* or Madagascar or Malagasy* or Malawi* or Mali* or Mozambiqu* or Nepal* or Niger* or Rwanda* or Senegal* or "Sierra Leon*" or Somalia* or "South Sudan*" or Tanzania* or Togo* or Uganda* or Zimbabw*)) OR AB ((afghan* or Benin* or Burkin* or Burundi* or "Central African Republic*" or Chad or chadian or Comoros or Comorian or Congo* or Eritrea* or Ethiopia* or Gambia* or Guinea* or Haiti* or "North Korea*" or "Democratic People's Republic of Korea" or Liberia* or Madagascar or Malagasy* or Malawi* or Mali* or Mozambiqu* or Nepal* or Niger* or Rwanda* or Senegal* or "Sierra Leon*" or Somalia* or "South Sudan*" or Tanzania* or Togo* or Uganda* or Zimbabw*))	154,937
S4	TI (("third world" or "less developed" or developing or "under developed" or underdeveloped or "middle income" or "low* income" or "less* developed" or "under served" or underserved or poor* or deprived) N3 (countr* or world or population* or nation* or gross domestic or	50,696

	gross national or gdp or gmp or economics or economy or economies)) OR AB (("third world" or "less developed" or developing or "under developed" or underdeveloped or "middle income" or "low* income" or "less* developed" or "under served" or underserved or poor* or deprived) N3 (countr* or world or population* or nation* or gross domestic or gross national or gdp or gmp or economics or economy or economies))	
S3	TI ((lami or lic or lics) N3 countr*)) OR AB ((lami or lic or lics) N3 countr*)) OR TI (lmic or lmics) OR AB (lmic or lmics)	3,706
S2	(MH "Continuous Positive Airway Pressure") OR TI ("continuous positive airway pressure" or bcpap or cpap or nippv or "non-invasive positive pressure ventilation") OR AB ("continuous positive airway pressure" or bcpap or cpap or nippv or "non-invasive positive pressure ventilation")	8,230
S1	((MH "Child+") OR (MH "Infant+")) OR TI PICU OR TI (infant* or baby or babies or toddler* or pediatric* or paediatric*) OR AB (infant* or baby or babies or toddler* or pediatric* or paediatric*)	871,705

Web of Science Core (Clarivate)

8	#7 AND #1 AND #2	362
7	#3 OR #4 OR #5 OR #6	6,879,627
6	TS=(afghan* or Benin* or Burkin* or Burundi* or "Central African Republic*" or Chad or chadian or Comoros or Comorian or Congo* or Eritrea* or Ethiopia* or Gambia* or Guinea* or Haiti* or "North Korea*" or "Democratic People's Republic of Korea" or Liberia* or Madagascar or Malagasy* or Malawi* or Mali* or Mozambiqu* or Nepal* or Niger* or Rwanda* or Senegal* or "Sierra Leon*" or Somalia* or "South Sudan*" or Tanzania* or Togo* or Uganda* or Zimbabw* or Angola* or Armenia* or Bangladesh* or Bhutan* or Bolivia* or "Cabo Verd*" or Cambodia* or Cameroon* or Congo* or "Cote d'Ivoir*" or Djibout* or Egypt* or "El Salvador*" or Georgia* or Ghana* or Guatemala* or Hondura* or India* or Indonesia* or Jordan* or Kenya* or Kiribati or Kosov* or Kyrgyz* or Laos or laotian or "lao pdr" or Lesoth* or Mauritania* or Micronesia* or Moldov* or Mongolia* or Morocc* or Myanmar* or Burmese or Nicaragua* or Nigeria* or Pakistan* or Papua* or Philippin* or "Sao Tome*" or "Solomon Island*" or "Sri Lank*" or Sudan* or Swazi* or ESWATINI or Syria* or Tajik* or Timor* or Tunisia* or Ukrain* or Uzbek* or Vanuatu* or Vietnam* or "West Bank" or Gaza or palestina* or Yemen* or Zambia* or Albania* or Algeria* or Samoa* or Argentina* or Azerbaijan* or Belarus* or Belize or Bosnia* or Herzegovina* or Botswana* or Brazil* or Bulgaria* or Chinese or China or Colombia* or "Costa Rica*" or Croatia* or Cuba* or Dominica* or Guinea* or Ecuador* or Fiji* or Gabon* or Grenada* or Guyana* or Iran* or Iraq* or Jamaica* or Kazakh* or Lebanon or lebanese or Libya* or Macedonia* or Malaysia* or Maldiv* or "Marshall Island*" or Mauriti* or Mexic* or Montenegr* or Namibia* or Nauru* or Panama* or Paraguay* or Peru* or Romania* or Russia* or Samoa* or Serbia* or "South Africa*" or Lucia* or "St Vincent" or Grenadin* or Suriname* or Thailand or thai or Tonga* or Turkey or TURKIYE or turkish or Turkmen* or Tuvalu* or Venezuela* or PALAU or Belau or pelew)	5,940,205
5	TI=(("third world" or "less developed" or developing or "under developed" or underdeveloped or "middle income" or "low* income" or "less* developed" or "under served" or underserved or poor* or deprived) AND (countr* or world or population* or nation* or gross domestic or gross national or gdp or gmp or economics or economy or economies)) OR AB=(("third world" or "less developed" or developing or "under developed" or underdeveloped or "middle income" or "low* income" or "less* developed" or "under served" or underserved or poor* or deprived) AND (countr* or world or population* or nation* or gross domestic or gross national or gdp or gmp or economics or economy or economies))	1,306,402
4	TI=(lmic or lmics) OR AB=(lmic or lmics)	9,457
3	TS=((lami or lic or lics) NEAR/3 countr*)	493
2	TS=("continuous positive airway pressure" or bcpap or cpap or nippv or "non-invasive positive pressure ventilation")	19,370
1	TS=(infant* or baby or babies or toddler* or pediatric* or paediatric*)	1,056,793

Appendix 3.2 Search strategy for grey literature.

On July 18, 2023, to identify grey literature, we conducted Google searches of the websites of relevant multinational and government agencies, non-governmental organizations, and healthcare facilities identified as bCPAP implementers in our peer-reviewed literature search. We targeted specific multinational and government agencies and non-governmental organizations identified by our core review

team and stakeholder advisory panel as relevant to our research question. We identified 30 specific healthcare facilities that had implemented bCPAP from the peer-reviewed literature and identified websites for nine facilities. Lastly, we searched the ministry of health websites of all low- and middle-income countries with identifiable websites.

We used the following Google search strategy. Non-English language sites were searched using the Google translate tool.

Google search strategy:

lmic|lmics|"low middle income" infant|infants|baby|babies|toddler|toddlers|pediatric|pediatrics|paediatric|paediatrics "continuous positive airway pressure" OR bcpap OR cpap OR nippv OR "non-invasive positive pressure ventilation"

Multinational agency and non-governmental organization sites searched for grey literature

Organization	Website
World Health Organization	https://www.who.int/
UNICEF	https://www.unicef.org/
Save the Children	https://www.savethechildren.net/
Stop Pneumonia/Every Breath Counts	https://stoppneumonia.org/
Essential Emergency and Critical Care Network	https://eeccnetwork.org/
Child Health Task Force	https://www.childhealthtaskforce.org/
Bill and Melinda Gates Foundation	https://www.gatesfoundation.org/
World Bank	https://www.worldbank.org/
PATH	https://www.path.org/
Clinton Health Action Initiative	https://www.clintonhealthaccess.org/
The Global Fund	https://www.theglobalfund.org/en/
Doctors without Borders	https://www.doctorswithoutborders.org/
Project C.U.R.E.	https://projectcure.org/
Afya Foundation	https://afyafoundation.org/
MedShare	https://www.medshare.org/
World Medical Relief	https://www.worldmedicalrelief.org/
International Medical Relief	https://internationalmedicalrelief.org/
Not Just Tourists	https://njt.net/
U.S. Agency for International Development	https://www.usaid.gov/

bCPAP implementers from peer-reviewed literature with identifiable websites searched grey literature

Facility	Website	Reference
Gulu Regional Hospital, Uganda	https://guluhospital.net/	Bjorklund et al., 2019
Red Cross War Memorial Children's Hospital, South Africa	https://www.childrenshospitaltrust.org.za/the-hospital/	Browde et al., 2019
International Centre for Diarrhoeal Disease Research, Bangladesh	https://www.icddrb.org/	Chisti et al., 2015
Hawassa District Hospital, Ethiopia		Gebre et al., 2022
Durame District Hospital, Ethiopia		
Wolisso District Hospital, Ethiopia		
Worabe District Hospital, Ethiopia		
Dil Chora District Hospital, Ethiopia		
Sabian District Hospital, Ethiopia		
Batu District Hospital, Ethiopia		

Tulubolo District Hospital, Ethiopia		
Fitche District Hospital, Ethiopia		
Butajira District Hospital, Ethiopia		
Tirunesh Beijing District Hospital, Ethiopia		
Shegaw Motta District Hospital, Ethiopia		
Yekatit 12 Hospital Medical College, Ethiopia		
St. Paul's Millenium Medical College, Ethiopia	https://sphmmc.edu.et/	
Butajira hospital, Ethiopia		
Bishoftu hospital, Ethiopia	https://bgh.gov.et/index.php/en/	
Queen Elizabeth Central Hospital, Malawi	https://qech.health.gov.mw/	Gondwe et al., 2017; Machen et al., 2015
St. Stephen's Hospital (SSH), Delhi, India	https://www.ststephenshospital.org/	Lal et al., 2016
Magunga District Hospital, Korogwe, Tanzania		Larsen & Poulsen, 2020
Salima District Hospital, Salima, Malawi		
Kamuzu Central Hospital, Lilongwe, Malawi		
Port Moresby General Hospital, Papua New Guinea	https://www.pomgen.gov.pg/	Pulsan et al., 2019
Dayanand Medical College and Hospital, Ludhiana, India	https://www.dmch.edu/	Punn et al., 2022
Mampong District Hospital, Ghana		Wilson et al., 2013
Kintampo Municipal Hospital, Ghana		
Nkoranza District Hospital, Ghana		
Wenchi District Hospital, Ghana		

National Ministry of Health websites searched for grey literature

**Country**	**Ministry of Health website**
Afghanistan	https://moph.gov.af/en
Albania	https://shendetesia.gov.al/
Argentina	https://www.argentina.gob.ar/salud
Armenia	https://www.moh.am/#1/0
Azerbaijan	https://sehiyye.gov.az/
Bangladesh	http://www.mohfw.gov.bd/
Belarus	https://minzdrav.gov.by/
Belize	https://www.health.gov.bz/
Benin	https://sante.gouv.bj/
Bhutan	https://www.moh.gov.bt/
Bolivia	https://www.minsalud.gob.bo/
Bosnia and Herzegovina	http://www.fbihvlada.gov.ba/
Brazil	https://www.gov.br/saude/pt-br
Bulgaria	https://www.mh.government.bg/bg/
Burkina Faso	https://www.sante.gov.bf/accueil
Burundi	http://minisante.bi/
Cabo Verde	https://minsaude.gov.cv/
Cambodia	http://moh.gov.kh/

Cameroon	https://www.minsante.cm/
China	http://en.nhc.gov.cn/
Colombia	
Congo	https://sante.gouv.cg/
Costa Rica	https://www.ministeriodesalud.go.cr/
Cuba	https://salud.msp.gob.cu/
DRC	https://www.minisanterdc.cd/
Cote d'ivoire	https://www.sante.gouv.ci/
Djibouti	https://sante.gouv.dj/
Dominican Republic	https://www.msp.gob.do/web/
Ecuador	https://www.salud.gob.ec/
Egypt	
El Salvador	https://www.salud.gob.sv/
Equatorial Guinea	https://guineasalud.org/
Ethiopia	https://www.moh.gov.et/site/
Fiji	https://www.health.gov.fj/
Gabon	https://www.sante.gouv.ga/
Georgia	https://www.moh.gov.ge/
Ghana	https://www.moh.gov.gh/
Guatemala	https://www.mspas.gob.gt/
Guinea	https://sante.gov.gn/
Haiti	https://www.mspp.gouv.ht/
Honduras	https://www.salud.gob.hn/sshome/
India	https://main.mohfw.gov.in/
Indonesia	https://www.kemkes.go.id/
Iran	https://irangov.ir/ministry-of-health-and-medical-education
Jamaica	https://www.moh.gov.jm/
Jordan	https://moh.gov.jo/Default/En
Kazakhstan	https://www.gov.kz/memleket/entities/dsm?lang=en
Kenya	https://www.health.go.ke/
Kiribati	https://mhms.gov.ki/
Lebanon	https://www.moph.gov.lb/
Lesotho	https://www.gov.ls/
Liberia	http://moh.gov.lr/
Libya	https://csc.gov.ly/en/portfolio/ministry-of-health/
Malaysia	https://www.moh.gov.my/
Maldives	https://health.gov.mv/dv
Mali	http://www.sante.gov.ml/
Malawi	https://www.health.gov.mw/
Mauritania	https://www.sante.gov.mr/
Mauritius	https://health.govmu.org/Pages/default.aspx
Mexico	https://www.gob.mx/salud

Micronesia	https://hsa.gov.fm/
Moldova	https://ms.gov.md/
Mongolia	https://moh.gov.mn/
Montenegro	https://www.gov.me/mzd
Morocco	https://www.sante.gov.ma/Pages/Accueil.aspx
Mozambique	https://www.misau.gov.mz/
Myanmar	https://www.mohs.gov.mm/
Namibia	https://mhss.gov.na/
Nepal	https://mohp.gov.np/np
Nicaragua	https://www.minsa.gob.ni/
Niger	https://www.sante.gouvne.org/
Nigeria	https://www.health.gov.ng/
North Korea	http://www.moph.gov.kp/en/
Pakistan	https://nhsrc.gov.pk/
Palau	https://www.palauhealth.org/
Palestine	https://www.moh.gov.ps/portal/
Papua New Guinea	http://www.health.gov.pg/
North Macedonia	https://zdravstvo.gov.mk/
Paraguay	https://www.mspbs.gov.py/index.php
Peru	https://www.gob.pe/minsa/
Philippines	https://doh.gov.ph/
Russian Federation	https://minzdrav.gov.ru/
Rwanda	https://www.moh.gov.rw/
Samoa	http://www.samoagovt.ws/tag/ministry-of-health/
Serbia	https://www.zdravlje.gov.rs/
Sierra Leone	https://mohs.gov.sl/
Solomon Islands	https://solomons.gov.sb/ministry-of-health-medical-services/
Somalia	https://moh.gov.so/en/
South Africa	https://www.health.gov.za/
South Sudan	https://moh.gov.ss/
Sri Lanka	http://www.health.gov.lk/
St Lucia	https://health.govt.lc/
St Vincent and the Grenadines	https://www.gov.vc/
Tanzania	https://www.moh.go.tz/index.php
Togo	https://sante.gouv.tg/
Tonga	http://www.health.gov.to/
Tunisia	http://www.santetunisie.rns.tn/fr/
Turkey	https://www.saglik.gov.tr/
Uganda	https://www.health.go.ug/
Ukraine	https://en.moz.gov.ua/
Uzbekistan	https://ssv.uz/
Vanuatu	https://moh.gov.vu/

Vietnam	https://moh.gov.vn/
Zambia	https://www.moh.gov.zm/
Zimbabwe	http://www.mohcc.gov.zw/

Appendix 4. Reasons for exclusion at full-text stage.

51 peer-reviewed references were excluded at the full-text stage, with reasons listed below.

Reference	Reason for exclusion
1. Alarcon J, Palacio S, Restrepo RD. Use of noninvasive ventilation in pediatric patients with pleural effusion associated with dengue virus infection: a retrospective review. Respiratory Care. 2016;61(10):OF40-OF.	Non-bCPAP device
2. Amadi HO, Okonkwo IR, Abioye IO, Abubakar AL, Olateju EK, Adesina CT, et al. A new low-cost commercial bubble CPAP (bCPAP) machine compared with a traditional bCPAP device in Nigeria. Paediatrics and international child health. 2019;39(3):184-92.	Neonatal/preterm (<28 days) or neonatal ICU population
3. Angurana SK, Takia L, Sarkar S, Jangra I, Bora I, Ratho RK, Jayashree M. Clinico-virological Profile, Intensive Care Needs, and Outcome of Infants with Acute Viral Bronchiolitis: A Prospective Observational Study. Indian journal of critical care medicine : peer-reviewed, official publication of Indian Society of Critical Care Medicine. 2021;25(11):1301-7.	Non-bCPAP device
4. Annankra WB, Mavis SC, Shany E, Jacob BM, Bakari A, Yeboah R, et al. Impact of an Online Program of Bubble Continuous Positive Airway Pressure Education in a Resource-Constrained Setting. ATS scholar. 2023;4(1):87-95.	Neonatal/preterm (<28 days) or neonatal ICU population
5. Bennett DJ, Carroll RW, Kacmarek RM. Evaluation of a Low-Cost Bubble CPAP System Designed for Resource-Limited Settings. Respiratory care. 2018;63(4):395-403.	Non-inpatient setting
6. Bhattacharya D, Dash N, Kavitha TK, Sharma M, Gautam V, Verma S. Lurking Infantile Pertussis: Experience from a Tertiary Care Center in Northern India. Journal of Pediatric Infectious Diseases. 2020;15(5):257-61.	bCPAP use only mentioned as outcome
7. Brown J, Machen H, Kawaza K, Mwanza Z, Iniguez S, Lang H, et al. A high-value, low-cost bubble continuous positive airway pressure system for low-resource settings: technical assessment and initial case reports. Plos One. 2013;8(1):e53622.	Non-inpatient setting
8. Buendia JA, Feliciano-Alfonso JE, Florez ID. Systematic review and cost-utility of high flow nasal cannula versus continuous positive airway pressure in children with acute severe or moderate bronchiolitis in Colombia. Pediatric pulmonology. 2022;57(12):3111-8.	Non-bCPAP device
9. C.R V, Sharma R, Jayashree M, Nallasamy K, Bansal A, Angurana SK, et al. Epidemiology, Clinical Profile, Intensive Care Needs and Outcome in Children with SARS-CoV-2 Infection Admitted to a Tertiary Hospital During the First and Second Waves of the COVID-19 Pandemic in India. Indian Journal of Pediatrics. 2022.	Non-bCPAP device
10. Cam BV, Tuan DT, Fonsmark L, Poulsen A, Tien NM, Tuan HM, Heegaard ED. Randomized comparison of oxygen mask treatment vs. nasal continuous positive airway pressure in dengue shock syndrome with acute respiratory failure. Journal of tropical pediatrics. 2002;48(6):335-9.	Non-bCPAP device
11. Carroll CL, Zucker AR. Barotrauma not related to type of positive pressure ventilation during severe asthma exacerbations in children. Journal of Asthma. 2008;45(5):421-4.	Non-bCPAP device
12. Cesar RG, Bispo BRP, Felix PHCA, Modolo MCC, Souza AAF, Horigoshi NK, Rotta AT. High-Flow Nasal Cannula versus Continuous Positive Airway Pressure in Critical Bronchiolitis: A Randomized Controlled Pilot. Journal of Pediatric Intensive Care. 2020;9(4):248-55.	Non-bCPAP device
13. Chaves GS, Freitas DA, Santino TA, Nogueira PAM, Fregonezi GA, Mendonca KM. Chest physiotherapy for pneumonia in children. The Cochrane database of systematic reviews. 2019;1:CD010277.	Review article
14. Crehan C, Colbourn T, Heys M, Molyneux E. Evaluation of 'TRY': an algorithm for neonatal continuous positive airways pressure in low-income settings. Archives of disease in childhood. 2018;103(8):732-8.	Neonatal/preterm (<28 days) or neonatal ICU population

15. Dada S, Ashworth H, Sobitschka A, Raguveer V, Sharma R, Hamilton RL, Burke T. Experiences with implementation of continuous positive airway pressure for neonates and infants in low-resource settings: A scoping review. Plos One. 2021;16(6):e0252718.	Review article
16. Ekhaguere OA, Mairami AB, Kirpalani H. Risk and benefits of Bubble Continuous Positive Airway Pressure for neonatal and childhood respiratory diseases in Low- and Middle-Income countries. Paediatric respiratory reviews. 2019;29:31-6.	Review article
17. Falk M, Donaldsson S, Drevhammar T. Correction: Infant CPAP for low-income countries: An experimental comparison of standard bubble CPAP and the Pumani system (PLoS ONE (2018) 13:5 (e0196683) DOI: 10.1371/journal.pone.0196683). Plos One. 2018;13(7):e0201083.	Non-inpatient setting
18. Fan X, Pan J. Clinical effect of NCPAP in treatment of children with severe pneumonia and its impact on their cardiac function. Maternal and Child Health Care of China. 2015;30(33):5898-901.	Non-bCPAP device
19. Farre R, Gozal D, Nguyen V-N, Pearce JM, Dinh-Xuan AT. Open-Source Hardware May Address the Shortage in Medical Devices for Patients with Low-Income and Chronic Respiratory Diseases in Low-Resource Countries. Journal of personalized medicine. 2022;12(9).	Non-inpatient setting
20. Floersch J, Hauschildt E, Keester A, Poganski S, Tran K, Slusher T, et al. A Low-Resource Oxygen Blender Prototype for Use in Modified Bubble CPAP Circuits. Journal of Medical Devices, Transactions of the ASME. 2020;14(1):015001 EN.	Non-inpatient setting
21. Habib MI, Khan KMA. Profile and outcomes of critically ill children in a lower middle-income country. Emergency medicine journal : EMJ. 2018;35(1):52-5.	bCPAP use only mentioned as outcome
22. Hansmann A, Morrow BM, Lang HJ. Review of supplemental oxygen and respiratory support for paediatric emergency care in sub-Saharan Africa. African Journal of Emergency Medicine. 2017;7:S10-S9.	Review article
23. Huang C, Wang P, Meng L, Li X, Rao F, Lu S. To study the treatment effect of mechanical expectoration cooperate with NCPAP on light and medium respiratory failure caused by severe pneumonia in infants and young children. Journal of Tropical Medicine (Guangzhou). 2014;14(12):1591-3.	Non-bCPAP device
24. Inglis R, Ayebale E, Schultz MJ. Optimizing respiratory management in resource-limited settings. Current opinion in critical care. 2019;25(1):45-53.	Review article
26. Jat KR, Dsouza JM, Mathew JL. Continuous positive airway pressure (CPAP) for acute bronchiolitis in children. The Cochrane database of systematic reviews. 2022;4:CD010473.	Review article
27. Lee H, Choi S, Park JY, Jo DS, Choi UY, Lee H, et al. Analysis of Critical COVID-19 Cases Among Children in Korea. Journal of Korean Medical Science. 2022;37(1):10.	Non-bCPAP device
28. Lin J, Zhang Y, Xiong L, Liu S, Gong C, Dai J. High-flow nasal cannula therapy for children with bronchiolitis: a systematic review and meta-analysis. Archives of disease in childhood. 2019;104(6):564-76.	Non-bCPAP device
29. Liu J, Wang Q, Qian SY, Xu WM, Li LL, Ning LM, et al. [Nasal continuous positive airway pressure ventilation in children with community-acquired pneumonia under five years of age: a prospective, multi-center clinical study]. Zhonghua er ke za zhi = Chinese journal of pediatrics. 2017;55(5):329-33.	Non-bCPAP device
30. Luo Y. Assessment of blood gas analysis results and degree of infection in children with severe pneumonia and respiratory failure after NCPAP therapy. Journal of Hainan Medical University. 2016;22(18):2118-21.	Non-bCPAP device
31. Morales MSL, Rodriguez ALDLR, Pascual JCR, Porras ON, Alvarez BAN. Efficiency of non-invasive mechanic ventilation in pediatric patients with acute respiratory failure. Revista del Instituto Nacional de Enfermedades Respiratorias. 2004;17(3):181-91.	Non-bCPAP device
32. Morrow BM, Feldman C, Green RJ. Acute viral bronchiolitis in South Africa: Intensive care management for severe disease. Samj South African Medical Journal. 2016;106(5):32-4.	Non-bCPAP device
33. Neighbour R, Eltringham R, Reynolds C, Meek J. Affordable CPAP in low income countries. Update in Anaesthesia. 2016;31:63-5.	Non-inpatient setting

34. Nguyen The Nguyen P, Trang Thi Kieu P, Diep Tuan T. Respiratory distress associated with dengue hemorrhagic fever on paediatric patients: learning from a provincial hospital in southern Vietnam. Archives of Pharmacy Practice. 2019;10(3):92-7.	Non-bCPAP device
35. Nielsen KR, Becerra MR, Mallma G, Ellington LE, Onchiri F, Roberts JS, et al. Nasal high flow therapy introduction lowers reintubation risk in a Peruvian paediatric intensive care unit. Acta paediatrica (Oslo, Norway : 1992). 2020;109(12):2748-54.	Non-bCPAP device
36. Ning J. Diagnosis and treatment of children with severe influenza A from the 2009/2010 pandemic in Tianjin, China. Tropical doctor. 2014;44(2):69-70.	Non-bCPAP device
37. Norgaard M, Stagstrup C, Lund S, Poulsen A. To Bubble or Not? A Systematic Review of Bubble Continuous Positive Airway Pressure in Children in Low- and Middle-Income Countries. Journal of tropical pediatrics. 2020;66(3):339-53.	Review article
Ongun EA, Dursun O, Anil AB, Altug U, Temel Koksoy O, Akyildiz BN, et al. A multicentered study on efficiency of noninvasive ventilation procedures (SAFE-NIV). Turkish journal of medical sciences. 2021;51(3):1159-71.	Non-bCPAP device
38. Orzechowska I, Sadique MZ, Thomas K, Davis P, Morris KP, Mouncey PR, et al. First-line support for assistance in breathing in children: statistical and health economic analysis plan for the FIRST-ABC trial. Trials. 2020;21(1):9.	Non-bCPAP device
39. Poletto S, Trevisanuto D, Ramaswamy VV, Seni AHA, Ouedraogo P, Dellaca R, Zannin E. Bubble CPAP respiratory support devices for infants in low-resource settings. Pediatric pulmonology. 2022.	Review article
40. Schlueter D, Kovaleski C, Walter V, Thomas NJ, Krawiec C. Impact of Body Mass Index and Initial Respiratory Support on Pediatric Subjects in Acute Respiratory Failure. Respiratory Care. 2021;66(9):1425-32.	Non-bCPAP device
41. Sessions KL, Smith AG, Holmberg PJ, Wahl B, Mvalo T, Chisti MJ, et al. Continuous positive airway pressure for children in resource-limited settings, effect on mortality and adverse events: systematic review and meta-analysis. Archives of disease in childhood. 2022;107(6):543-52.	Review article
42. Simkovich SM, Underhill LJ, Kirby MA, Crocker ME, Goodman D, McCracken JP, et al. Resources and Geographic Access to Care for Severe Pediatric Pneumonia in Four Resource-limited Settings. American Journal of Respiratory and Critical Care Medicine. 2022;205(2):183-97.	Non-bCPAP device
43. Van Cam B, Grytter C, Tuan DT. Nasal continuous positive airway-pressure treatment for pneumonia in Vietnam. Lancet (London, England). 1995;345(8950):656.	Non-bCPAP device
44. Verma N, Lodha R, Kabra SK. Recent advances in management of bronchiolitis. Indian Pediatrics. 2013;50(10):939-49.	Non-bCPAP device
45. Vincent CA, Mboga M, Gathara D, Were F, Amalberti R, English M. How to do no harm: empowering local leaders to make care safer in low-resource settings. Archives of disease in childhood. 2021;106(4):333-7.	Non-bCPAP device
46. Wilkes C, Subhi R, Graham HR, Duke T, group ARIR. Continuous Positive Airway Pressure (CPAP) for severe pneumonia in low- and middle-income countries: A systematic review of contextual factors. Journal of global health. 2022;12:10012.	Review article
47. Won A, Suarez-Rebling D, Baker AL, Burke TF, Nelson BD. Bubble CPAP devices for infants and children in resource-limited settings: review of the literature. Paediatrics and international child health. 2019;39(3):168-76.	Review article
48. Wu A, Mukhtar-Yola M, Luch S, John S, Adhikari BR, Bakker C, et al. Innovations and adaptations in neonatal and pediatric respiratory care for resource constrained settings. Frontiers in pediatrics. 2022;10:954975.	Review article
49. Zhang X, Yao J, Gao L, Chen J, Li F. Emergency treatment of acute severe pneumonia children complicated with respiratory failure and etiological analysis. Chinese Journal of Nosocomiology. 2019;29(6):941-4.	Non-bCPAP device

50. Zhang Y, Ruan L, Wang Z, Zhang C, Zhang G. Clinical treatment of children with severe pneumonia and respiratory failure pathogens causing VAP infection and risk factors analysis. Chinese Journal of Nosocomiology. 2019;29(7):1102-5.	Non-bCPAP device
51. Zheng Y-R, Lin W-H, Lin S-H, Xu N, Cao H, Chen Q. Bi-level Positive Airway Pressure Versus Nasal CPAP for the Prevention of Extubation Failure in Infants After Cardiac Surgery. Respiratory care. 2022;67(4):448-54.	Non-bCPAP device

Appendix 5. Assessments of rigor for included studies, by study type.

We used Critical Appraisal Skills Programme (CASP) checklists for randomized trials, cohort studies, economic evaluations, and qualitative studies. References that did not fit any of these study types were assessed for relevance and rigor as described in the main manuscript.

Appendix 5.1 Appraisals of randomized, controlled trials and protocols (n=7).

	1. Clearly focused research question?	2. Assignment randomized?	3. All enrolled participants accounted for at conclusion?	4a. Participants blinded?	4b. Investigators blinded?	4c. People assessing outcomes blinded??	5. Study groups similar at start of trial?	6. Study groups received same level of care?	7. Intervention effects reported comprehensively?	8. Precision of intervention effect estimate reported?	9. Benefits of intervention outweigh harms and costs?	10. Results can be applied to children aged 1-59m in LMIC contexts?	11. Intervention provides greater value than other existing LMIC interventions?	
Chisti et al. (2015), Bangladesh	Y	Y	Y	N	N	N	Y	Y	Y	Y	Y	Y	Y	
Gebre et al. (2022), Ethiopia	Y	Y	NA	N	N	N	NA	NA	NA	NA	NA	NA	NA	
Lal et al. (2016), India	Y	Y	Y	N	N	N	Unknown	Unknown	N	N	Unknown	Y	Unknown	
McCollum et al. (2019), Malawi	Y	Y	Y	N	N	N	Y	Y	Y	Y	N	Y	N	
Smith et al. (2017), Malawi	Y	Y	NA	N	N	N	NA	NA	NA	NA	NA	NA	NA	
Wilson et al. (2013), Ghana	Y	Y	Y	N	N	N	Y	Y	Y	Y	Y	Y	Y	
Wilson et al. (2017), Ghana	Y	Y	Y	N	N	N	Y	Y	Y	Y	Y	Y	Y	

Appendix 5.2 Appraisals of pre-post interventional trials and quantitative observational studies (n=12).

Each of the following tables represents the CASP checklist appraisal for one study.

**Authors (year), country**	**Bjorklund et al. (2019), Uganda**
**1. Clearly focused issue?**	Y
**2. Cohort recruitment acceptable?**	Y
**3. Exposure accurately measured to minimize bias?**	Y
**4. Outcome accurately measured to minimize bias?**	Y
**5a. All important confounders identified?**	N
**5b. Confounders accounted for in design and/or analysis?**	N
**6a. Was subject follow up complete enough?**	Y
**6b. Was subject follow up long enough?**	Y
**7. What are the results?**	There were no significant complications related to SEAL-bCPAP use. 4 out of 83 patients developed mild complications that resolved after SEAL-bCPAP removal or nasogastric tube placement.
**8. How precise are the results?**	Study not powered to assess efficacy.
**9. Do you believe the results?**	Y
**10. Results can be applied to children aged 1-59m in LMIC contexts?**	Y
**11. Do the results fit with other available evidence?**	Y
**12. What are the implications?**	SEAL-bCPAP is a safe mode of respiratory support in children aged 1 month to 5 years. The study was not powered to assess efficacy.

**Authors (year), country**	**Browde et al. (2019), South Africa**
**1. Clearly focused issue?**	Y
**2. Cohort recruitment acceptable?**	Y
**3. Exposure accurately measured to minimize bias?**	Y

**4. Outcome accurately measured to minimize bias?**	Y
**5a. All important confounders identified?**	N
**5b. Confounders accounted for in design and/or analysis?**	N
**6a. Was subject follow up complete enough?**	Y
**6b. Was subject follow up long enough?**	Y
**7. What are the results?**	Five out of thirty-one patients (15.6%) failed NIV and were intubated and mechanically ventilated.
**8. How precise are the results?**	Unable to estimate prevalence of NIV use in this population due to small sample size. Unable to perform multivariate logistic regression for primary outcome due to rarity of event and small sample size.
**9. Do you believe the results?**	Y
**10. Results can be applied to children aged 1-59m in LMIC contexts?**	Y
**11. Do the results fit with other available evidence?**	Unknown
**12. What are the implications?**	In this small, observational study, most patients who received NIV did not require invasive mechanical ventilation.

**Authors (year), country**	**Buys et al. (2023), South Africa**
**1. Clearly focused issue?**	Y
**2. Cohort recruitment acceptable?**	Y
**3. Exposure accurately measured to minimize bias?**	Unknown
**4. Outcome accurately measured to minimize bias?**	Y
**5a. All important confounders identified?**	Y
**5b. Confounders accounted for in design and/or analysis?**	Y
**6a. Was subject follow up complete enough?**	Y
**6b. Was subject follow up long enough?**	Y
**7. What are the results?**	38 of 500 children (8%) treated with bCPAP required invasive mechanical ventilation.
**8. How precise are the results?**	Powered for a precision of within 3% of estimate, but did not attempt to calculate actual precision of results.

**9. Do you believe the results?**	Y
**10. Results can be applied to children aged 1-59m in LMIC contexts?**	Y
**11. Do the results fit with other available evidence?**	Y
**12. What are the implications?**	In this retrospective chart review study, most children initiated on bCPAP for undifferentiated respiratory distress did not require ICU admission or invasive mechanical ventilation.

**Authors (year), country**	**Jayashree et al. (2016), India**
**1. Clearly focused issue?**	Y
**2. Cohort recruitment acceptable?**	Y
**3. Exposure accurately measured to minimize bias?**	Y
**4. Outcome accurately measured to minimize bias?**	Y
**5a. All important confounders identified?**	Y
**5b. Confounders accounted for in design and/or analysis?**	Y
**6a. Was subject follow up complete enough?**	Y
**6b. Was subject follow up long enough?**	Y
**7. What are the results?**	3 out of 163 infants (1.8%) on bCPAP for hypoxemic clinical pneumonia required intubation.
**8. How precise are the results?**	They appear to be precise because the study enrolled all consecutive patients for a year, but no precision calculations are reported.
**9. Do you believe the results?**	Y
**10. Results can be applied to children aged 1-59m in LMIC contexts?**	Y
**11. Do the results fit with other available evidence?**	No, other similar studies generally report much higher rates of bCPAP failure. Importantly, 9 of 16 patients intubated immediately at presentation died, and these patients may have been started on bCPAP in other studies.
**12. What are the implications?**	bCPAP is safe and potentially effective in children with hypoxemic clinical pneumonia.

**Authors (year), country**	**Kinikar et al. (2011), India**
**1. Clearly focused issue?**	Y

**2. Cohort recruitment acceptable?**	Y
**3. Exposure accurately measured to minimize bias?**	Y
**4. Outcome accurately measured to minimize bias?**	Y
**5a. All important confounders identified?**	Y
**5b. Confounders accounted for in design and/or analysis?**	Y
**6a. Was subject follow up complete enough?**	Y
**6b. Was subject follow up long enough?**	No, 6 hours may not be long enough to determine clinical outcomes associated with bCPAP use for pneumonia
**7. What are the results?**	Respiratory rate improved significantly after 6 hours of bCPAP treatment (from 65 breaths per minute to 45 breaths more minute)
**8. How precise are the results?**	Authors report p<.001 for the primary outcome.
**9. Do you believe the results?**	Y
**10. Results can be applied to children aged 1-59m in LMIC contexts?**	Y
**11. Do the results fit with other available evidence?**	Y
**12. What are the implications?**	bCPAP use may improve respiratory physiologic parameters within several hours of use.

Authors (year), country	**Machen et al. (2015), Malawi**
**1. Clearly focused issue?**	Y
**2. Cohort recruitment acceptable?**	Y
**3. Exposure accurately measured to minimize bias?**	N
**4. Outcome accurately measured to minimize bias?**	Y
**5a. All important confounders identified?**	N
**5b. Confounders accounted for in design and/or analysis?**	N
**6a. Was subject follow up complete enough?**	Y
**6b. Was subject follow up long enough?**	N
**7. What are the results?**	Respiratory Index of Severity in Children score decreased 24 hours after initiation of bCPAP, although effect size and precision are not reported.

**8. How precise are the results?**	Unknown, precision of estimates not reported
**9. Do you believe the results?**	Y
**10. Results can be applied to children aged 1-59m in LMIC contexts?**	Y
**11. Do the results fit with other available evidence?**	Y
**12. What are the implications?**	Because this study is does not address confounders or report effect size or precision, it is difficult to draw conclusions based on this study alone.

**Authors (year), country**	**Myers et al. (2018), Malawi**
**1. Clearly focused issue?**	Y
**2. Cohort recruitment acceptable?**	Y
**3. Exposure accurately measured to minimize bias?**	Y
**4. Outcome accurately measured to minimize bias?**	Y
**5a. All important confounders identified?**	N
**5b. Confounders accounted for in design and/or analysis?**	N
**6a. Was subject follow up complete enough?**	Y
**6b. Was subject follow up long enough?**	Y
**7. What are the results?**	Survival was lower in bCPAP patients with multiple organ failure, severe malnutrition, and HIV infection or exposure.
**8. How precise are the results?**	Odds ratios from univariate logistic regression reported and demonstrate precise estimates.
**9. Do you believe the results?**	Y
**10. Results can be applied to children aged 1-59m in LMIC contexts?**	Y
**11. Do the results fit with other available evidence?**	Y
**12. What are the implications?**	Children with severe pneumonia requiring bCPAP in certain subgroups (multiple organ failure, severe malnutrition, HIV infection or exposure) are at higher risk of death.

**Authors (year), country**	**Olayo et al. (2019), Kenya**
**1. Clearly focused issue?**	Y

**2. Cohort recruitment acceptable?**	Y
**3. Exposure accurately measured to minimize bias?**	Y
**4. Outcome accurately measured to minimize bias?**	Y
**5a. All important confounders identified?**	N
**5b. Confounders accounted for in design and/or analysis?**	N
**6a. Was subject follow up complete enough?**	Y
**6b. Was subject follow up long enough?**	Y
**7. What are the results?**	There were no statistically significant differences between first- and second-generation healthcare providers' bCPAP skills or knowledge scores.
**8. How precise are the results?**	Skills scores: 90% (95% CI 87-93%) vs 89% (95% CI 86-92%)<br>Knowledge scores: 91% (95% 88-93%) vs 90% (95% CI 88-93%)
**9. Do you believe the results?**	Y
**10. Results can be applied to children aged 1-59m in LMIC contexts?**	Y
**11. Do the results fit with other available evidence?**	Y
**12. What are the implications?**	Training of the trainers can be an effective model for introducing bCPAP in LMICs.

**Authors (year), country**	**Pulsan et al. (2019), Papua New Guinea**
**1. Clearly focused issue?**	Y
**2. Cohort recruitment acceptable?**	Y
**3. Exposure accurately measured to minimize bias?**	Y
**4. Outcome accurately measured to minimize bias?**	Y
**5a. All important confounders identified?**	N
**5b. Confounders accounted for in design and/or analysis?**	N
**6a. Was subject follow up complete enough?**	Y
**6b. Was subject follow up long enough?**	Y

**7. What are the results?**	A higher SpO2 at 1 hour after bCPAP initiation was a predictor of survival and HIV infection was a predictor of death.
**8. How precise are the results?**	Univariate logistic regression odds ratio for death comparing patients with HIV to those without: 12.8 (95% CI 1.5-106.8).
**9. Do you believe the results?**	Y
**10. Results can be applied to children aged 1-59m in LMIC contexts?**	Y
**11. Do the results fit with other available evidence?**	Y
**12. What are the implications?**	This study had a high mortality among children with severe pneumonia on bCPAP, especially in subgroups with comorbidities, implying that bCPAP use requires careful patient selection and implementation.

**Authors (year), country**	**Punn et al. (2022), India**
**1. Clearly focused issue?**	Y
**2. Cohort recruitment acceptable?**	Y
**3. Exposure accurately measured to minimize bias?**	No, all types of non-invasive ventilation (including bCPAP and bilevel airway pressure) included without separation. Only 34.8% of patients in this study received bCPAP; others received bilevel airway pressure.
**4. Outcome accurately measured to minimize bias?**	Y
**5a. All important confounders identified?**	N
**5b. Confounders accounted for in design and/or analysis?**	N
**6a. Was subject follow up complete enough?**	Y
**6b. Was subject follow up long enough?**	Y
**7. What are the results?**	81.7% of patients were successfully treated by non-invasive ventilation, while 18.3% failed and required intubation.
**8. How precise are the results?**	Not reported
**9. Do you believe the results?**	Y
**10. Results can be applied to children aged 1-59m in LMIC contexts?**	Y
**11. Do the results fit with other available evidence?**	Unknown
**12. What are the implications?**	Non-invasive ventilation may be an effective way to prevent intubation in children with respiratory failure, but further testing of this hypothesis is necessary.

**Authors (year), country**	**Sessions et al. (2019), Malawi**
**1. Clearly focused issue?**	Y
**2. Cohort recruitment acceptable?**	Y
**3. Exposure accurately measured to minimize bias?**	Y
**4. Outcome accurately measured to minimize bias?**	Y
**5a. All important confounders identified?**	Y
**5b. Confounders accounted for in design and/or analysis?**	Y
**6a. Was subject follow up complete enough?**	Y
**6b. Was subject follow up long enough?**	Y
**7. What are the results?**	Healthcare workers spent 12.45 minutes longer per patient setting up bCPAP equipment and 11.13 minutes longer setting up the bCPAP nasal interface compared to oxygen equipment and nasal cannula setup.
**8. How precise are the results?**	P<0.001 reported for both outcomes.
**9. Do you believe the results?**	Y
**10. Results can be applied to children aged 1-59m in LMIC contexts?**	Y
**11. Do the results fit with other available evidence?**	Unknown
**12. What are the implications?**	bCPAP implementation in LMICs will likely create additional healthcare worker burden relative to usual pneumonia care with oxygen.

**Authors (year), country**	**Walk et al. (2016), Malawi**
**1. Clearly focused issue?**	Y
**2. Cohort recruitment acceptable?**	Y
**3. Exposure accurately measured to minimize bias?**	Y
**4. Outcome accurately measured to minimize bias?**	Y
**5a. All important confounders identified?**	N
**5b. Confounders accounted for in design and/or analysis?**	N

**6a. Was subject follow up complete enough?**	Y
**6b. Was subject follow up long enough?**	Y
**7. What are the results?**	41 out of 77 children (53%) with acute respiratory failure survived following bCPAP treatment. Older children and HIV infected children had higher odds of bCPAP failure.
**8. How precise are the results?**	Not reported
**9. Do you believe the results?**	Y
**10. Results can be applied to children aged 1-59m in LMIC contexts?**	Y
**11. Do the results fit with other available evidence?**	Y
**12. What are the implications?**	While bCPAP implementation was feasible, patients receiving bCPAP, especially older children and HIV infected children, still exhibited high mortality.

Appendix 5.3 Appraisal of economic evaluation (n=1).

**Authors (year), country**	**Kortz et al. (2017), Malawi**
**1. Well-defined question?**	Y
**2. Comprehensive description of competing alternatives?**	Y
**3. Evidence that programme would be effective?**	Y
**4. Intervention effects identified, measured, and valued appropriately?**	Y
**5. All important resources and costs identified, measured, and valued credibly?**	Y
**6. Discounting?**	Y
**7. What were the results?**	The cost of bCPAP per patient was 15 USD per day and 41 USD per hospitalization. bCPAP averts 5.0 disability-adjusted life years (DALYs) per child treated, with an incremental cost-effectiveness ratio of 12.88 USD per DALY averted compared to standard of care.
**8. Was an incremental analysis of consequences and costs performed?**	Y
**9. Was an adequate sensitivity analysis performed?**	Y
**10. Is the program likely to be equally effective in LMIC settings?**	Y

**11. Are the costs translatable to LMIC settings?**	Y
**12. Is the intervention worth doing in LMIC settings?**	Y

Appendix 5.4 Appraisals of qualitative studies (n=5).

	1. Clear aims statement?	2. Is qualitative methodology appropriate?	3. Is the research design appropriate?	4. Recruitment strategy appropriate?	5. Was data collected in a way that addressed the research issue?	6. Has the relationship between researcher and participants been adequately considered?	7. Have ethical issues been considered?	8. Was data analysis sufficiently rigorous?	9. Clear statement of findings?	10. How valuable is the research?
Chisti et al. (2023)	Y	Y	Y	Y	Y	N	Y	Y	Y	Very valuable and relevant
Gebre et al. (2022)	Y	Y	Y	Y	Y	N	Y	Y	Y	Very valuable and relevant
Gondwe et al. (2017)	Y	Y	Y	Y	Y	Y	Y	Y	Y	Very valuable and relevant
Nyondo-Mipando et al. (2020)	Y	Y	Y	Y	Y	N	Y	Y	Y	Very valuable and relevant
Sessions et al. (2020)	Y	Y	Y	Y	Y	N	Y	Y	Y	Very valuable and relevant

Appendix 6. Final context-mechanism-outcome configurations with supporting and refuting evidence.

CMOC 1. In settings with limited financial resources (C), low-cost, locally made bCPAP devices that follow appropriate standards (R) build local expertise and resources (M), improving sustainability (O).

Supporting evidence	Refuting evidence	Supporting quotes	Refuting quotes
Chisti 2015: "The bubble CPAP system was constructed locally using standard nasal oxygen prongs (Ventlab, Mocksville, NC, USA), tubing used for administration of intravenous fluids (Opso Saline, Dhaka, Bangladesh), and a waterfilled, transparent shampoo bottle."<br><br>Duke 2014: "A relatively inexpensive bubble CPAP device can be made using low-resistance nasal oxygen prongs, or modified standard nasal oxygen prongs driven by an oxygen concentrator. [….] This system is being used in several hospitals in Asia (Dhaka Children's Hospital, Bangladesh and elsewhere) and Papua New Guinea, and is working effectively"<br><br>Gulla 2021: "Although India has now become a global market for many biomedical equipment and established itself as competitor for multinational counter parts, unfortunately hardly any of the NRS equipment or their parts are manufactured in India."<br><br>"To fill up the existing huge demand supply gap of non-invasive ventilation equipment, there is a need to develop high quality, locally manufactured, affordable non-invasive respiratory support equipment by facilitating partnership between governing agencies and industry."	Larsen 2021: "[…] we experienced that this homemade setup was very time demanding and the quality of the treatment critically depended on the clinician to monitor and constantly optimize the equipment."<br><br>Wilson 2014: "Although locally available equipment may help ensure that equipment can be serviced locally, it does not ensure durability, nor does it ensure proper care and use."<br><br>Buys 2023: "From a quality assurance point ofview, it is crucial that these devices follow internationally acceptable standards including the procurement and maintenance of equipment including the correct respiratory circuits, ensuring adequate flow in relation to the patient's size/weight."<br><br>PATH 2021: "bCPAP devices are one of the few medical devices that can plausibly be improvised from local materials. Given the high cost of commercial models, providers have a strong incentive to circumvent funding challenges and build a low-cost improvised device, despite those devices often being medically inferior (among other limitations, they typically do not heat or humidify the air being delivered). This creates a unique quality control challenge since the improvised devices are locally made and never pass through any centralized procurement or import process."<br><br>"At least a few low-cost and/or non-electric bCPAP prototypes have been developed and tested,	"I have seen many of these commercially made devices being thrown here and there because the parts are not available. […] The locally improvised [devices] to me outlast the commercially available ones […] because […] you have the parts." (Focus Group #2, Physician/Researcher, Ghana)<br><br>"[…] improvisation of the materials is very real; however there is quite concerning/valid [refuting] evidence." (Survey #1, Pediatric Nurse Practitioner, Malawi)<br><br>"Low-cost bubble CPAP devices need to follow standard characteristics [….] If no tested, good quality bCPAP device is available it may be better to use low-flow oxygen." (Survey #1, Pediatric Critical Care Physician/Researcher, Sub-Saharan Africa)<br><br>"Although it is true that locally made bCPAP […] might not provide adequate CPAP […], in some settings it has been found that if you have adequate amount of supervision with proper monitoring, it is as good as commercially made." (Focus Group #1, Pediatric Respiratory Physician, Bangladesh)<br><br>"I agree with the fact of promoting local manufacturing and/or innovations. However, quality assurance is more than food design or testing,	"In theory sure but in practice supply chains eventually fall apart or are disrupted and that severely impacts quality of care. Locally improvised devices end up creating permanent negative quality of life issues, 100% O2 delivered, or high infection rates come to mind."

Kortz 2017: "Our base case analysis demonstrated an ICER of $12.88 per DALY averted, which is highly cost-effective by most standards." [This cost-effectiveness study compared a locally manufactured bCPAP device to low-flow oxygen.]<br><br>Kinikar 2011: "The authors indigenously prepared a Nasal bubble CPAP circuit (Fig. 1) that could be easily set up at the patients' bedside and administer an end expiratory pressure of 5 cm of water with an FiO2 of 70% (confirmed on FiO2 analyzer); costing Rs 250/- only."<br><br>Chisti 2023: "It is a combination of three locally available devices: nasal canula, IV infusion set, and plastic bottle. It is prepared by combining one part with another. It is low cost, locally available and easy to prepare."<br><br>WHO 2016: "An inexpensive form of bubble CPAP can be made with standard nasal prongs. [….] This system is used in several hospitals in Asia (e.g. Dhaka Children's Hospital in Bangladesh)"	but none of them appears to be commercially available and the reasons why are not clear—none of the articles discuss any specific roadblocks or challenges to commercialization. Are there no manufacturers willing to take on production? Is country registration and/or regulatory approval a barrier? Are there certain capabilities the prototypes are missing (e.g., heated and humidified air) that clinicians are unwilling to compromise on? Further investigation is needed to help clarify these reasons."	it may be compatibility of materials, risks associated in case of break down, electrical risks, capacity to produce also spare parts, etc."	

CMOC 2. In settings with limited staffing (C), bCPAP devices and interfaces that are easy to use (R) facilitate HCWs[a] feeling confident in their training and ability (M), improving feasibility (O).

**Supporting evidence**	**Refuting evidence**	**Supporting quotes**	**Refuting quotes**
Bjorklund 2019: "bCPAP devices are ideal in LMIC because they […] are easy to assemble and do not require extensive training or monitoring"<br><br>Chisti et al. 2015: "Modified nasal oxygen prongs can be used, making it […] almost as easy for nurses to apply as standard flow oxygen therapy."			

Duke 2014: “CPAP using simple technology is likely to be of benefit […]”<br><br>Gebre 2022: “Therefore, they faced difficulty to estimate the true flow of oxygen […]”<br><br>Jayashree 2016: “Ease of administration and safety profile could make it a useful tool […]”<br><br>Larsen 2021: “[…] we experienced that this homemade setup was very time demanding and the quality of the treatment critically depended on the clinician to monitor and constantly optimize the equipment.”<br><br>Machen 2015: “The system is inexpensive and easy to repair, maintain, operate, and transport.”<br><br>McCollum 2011: “bCPAP requires little technical expertise […]”<br><br>Wilson 2014: “In response to open-ended questions, 16 of 28 [nurses] expressed a desire for more robust training [….] Eight nurses complained about the cumbersome nature of the CPAP set-up […].”			

CMOC 3. In settings with limited resources (C), advance budgeting for implementation costs (R) results in appropriate allocation of resources to bCPAP (M), improving feasibility and sustainability (O) of newly developed programs.

**Supporting evidence**	**Refuting evidence**	**Supporting quotes**	**Refuting quotes**
<u>Gulla 2021</u>: “In LMICs, availability and cost of interfaces are major hurdles to provide non-invasive respiratory support even in eligible children.”<br><br><u>Kortz 2017</u>: “For bCPAP, we also included the costs of provider training, pulse oximetry and NP suction as these are integral to the intervention.”	<u>André von-Arnim 2017</u>: “Change in workload, ongoing costs (electricity, consumables, and maintenance costs), re-usability and durability of equipment components, and initial equipment cost were rated less highly, even in LMIC.” [Also from the same paper: “The initial equipment cost was also not rated highly by participants, likely due to the strong representation of	“Limited resources are also linked to scarce skills and lack / few regulatory frameworks. The statement doesn't include other indirect costs like training, developing local clinical guidelines, implementing biomedical engineering workshops...”	

"We included the cost of essential capital equipment: an additional oxygen concentrator, pulse oximeter and NP suctioning device."<br><br>"The base case analysis shows that the cost of treating one child with severe pneumonia is $88 for standard of care and $152 for standard of care plus bCPAP"<br><br>Wilson 2013: "Further, even the cost of patient interfaces (nasal prongs $32) may be prohibitive in many cases. If CPAP is found to improve outcomes, efforts to develop low-cost alternatives to current CPAP machines and interfaces will become all the more critical."<br><br>Farré 2019: "There are three potential options to provide healthcare centers in LICs with the CPAP devices they cannot afford […]"<br><br>McCollum 2019: "The augmented clinical support might imply that similar LMIC district hospitals could not implement bCPAP without substantial investment"<br><br>Smith 2017: "For example, bCPAP devices, while not as costly as invasive methods of ventilation, are generally more expensive than traditional low-flow oxygen nasal cannula delivery."<br><br>PATH 2021: "Although bCPAP devices are generally cheaper than mechanical ventilator-based CPAP, the bCPAP models in PATH's 2014 Device Selection Guide still ranged from US$800-$6,000, which is a huge expense for many health facilities."	better-resourced healthcare facilities in both LMIC and HIC."]		

CMOC 4. In settings where bCPAP is introduced as a collaboration with external stakeholders (C), early post-study sustainability planning with local team members (R) creates opportunities for the longitudinal involvement of leaders with local influence (M), leading to improved equity and sustainability (O).

Supporting evidence	Refuting evidence	Supporting quotes	Refuting quotes
Baiden 2021: "Seven of eight CPAP machines that had been donated to the facilities after the trial were functional. However, five of eight oxygen concentrators and three of four electric generators that had been procured to facilitate the trial were non-functional."<br><br>"At the end of the trial, the CPAP machines, oxygen concentrators and generators were, upon request, donated to the two hospitals. In an unpublished assessment of the functionality of the equipment six years after the completion of the trial in 2015, we found that all eight CPAP machines and two oxygen generators were functional and still in use."<br><br>"While all the staff trained in Kintampo hospital were still at post, four out of the five staff trained at the Mampong Hospital had been transferred to other health facilities"<br><br>Morris 2014: "The effectiveness of a medical intervention is affected by the circumstances in which it is administered,18 and temporarily elevating the level of care in the context of a study may limit the extent to which the results of the study can be extrapolated to the real-world setting. The most relevant scientific question to the host community is the effectiveness of a therapy as delivered within the existing health care system."<br><br>"The CPAP machines and ancillary equipment were donated to the participating hospitals at the conclusion of the study. However, the challenges to fully integrating CPAP into medical practice in these district hospitals have not all been met. During the	Morris 2014: "Limitations on a requirement for post-trial availability are widely recognized, and a principled argument can be made to refute it entirely. Even proponents of the reasonable availability standard acknowledge that it may not be possible in all cases to ensure post-trial access, and the paucity of practical guidance has been noted."	"I don't necessarily agree that planning for sustainability alone results in leadership/empowerment. There are steps in between. It is more likely that that inclusion of local leadership/stakeholder buy-in in the early planning phase leads to empowerment and program sustainability." (Survey #2, Pediatric Nurse Practitioner, Malawi)<br><br>"There needs to be a degree of funding, which is […] integrated in local structures, the hospital, the Ministry of Health, including maintenance, logistic support, and supply chains." (Focus Group #2, Pediatric Critical Care Physician/Researcher, Sub-Saharan Africa)<br><br>"Supply chain comes to mind in 'local team leadership and empowerment of partnerships' or something to those lines threaded in the in these studies we open up avenues for purchasing/supplying and delivering devices that may be in their favor with a western partner involved early on but when we leave, or if prices/suppliers change, maybe that is implied, but how do they navigate that without the prestige of a western partner in tow, or even the extra bandwidth if we aren't adding a level of buying power/trust to negotiation with suppliers. Often we leave and supply lines dry up either in just the parts themselves and they cant navigate to find new suppliers, or, they lose a powerful ally in negotiations. I'd advocate for including that in your schematic not just in the paragraph supporting it."	

study, new tubing and IRB: Ethics & Human Research nasal interfaces were available for each research participant, and while a limited supply was left at each hospital, no funding exists for an indefinite supply of these items."<br><br>"The reasonable availability standard aims to decrease the potential for exploitation, stating that research should be undertaken in resource poor communities only when the product, if found to be effective, will be made reasonably available for benefit of that community. There are two separate aspects of the reasonable availability standard. The first is that individual subjects who enroll in a trial should have continuing access to the intervention if it is shown to be effective. The second is that an effective intervention should be available to the community in which the research took place."<br><br>"A plan for responsible transition to incorporating successful interventions into the health care system should be part of pre-trial planning and should occur in close consultation with stakeholders in the host country."<br><br><u>Farré 2019</u>: "In conclusion, we have effectively implemented a collaborative procedure for health technology transfer to a team in LICs, enabling autonomous construction and maintenance, thereby facilitating adequate provision of pediatric CPAP settings in markedly underserved regions. The approach is based on a concept that is usually overlooked when providing health technology support, namely, to empower the final users in LICs to fully control			

the procedure and adapt it to the specific local conditions.”<br><br>PATH 2021: “At least one study found that the vast majority of commercial bCPAP devices in use in Kenyan hospitals were donated. This high prevalence of donated devices led to concerns about long-term sustainability, training, and maintenance barriers, in line with the general medical device literature described previously.”			

CMOC 5. In settings with unreliable oxygen and power supply (C), ensuring reliable backup sources of oxygen and electricity (R) reduces disruptions in care and enhances HCWs’[a] perception of bCPAP safety (M), improving feasibility and fidelity (O).

**Supporting evidence**	**Refuting evidence**	**Supporting quotes**	**Refuting quotes**
Baiden 2021: “There has also been substantial improvement in oxygen and power supply in all four district hospitals that have been involved in the CPAP trials between 2011 and 2015. The continuous routine use of CPAP in these facilities does not require the special provision of oxygen concentrators and power supply.”<br><br>Chisti 2015: “These apparent limits of oxygen concentrator technology mean that a back-up source of oxygen or gas flow is needed for safe and effective bubble CPAP”<br><br>Duke 2014: “Introducing any method of CPAP is appropriate only when reliable systems for giving and monitoring oxygen therapy are in place, health workers are adequately trained and close monitoring is assured.”<br><br>Gebre 2022: “[…] the identified operational challenges were occasional lack of bubbling in the water-filled plastic bottle, lack of stand for holding the water-filled plastic bottle, and delayed shifting of oxygen source from an oxygen concentrator to a cylinder, particularly during electricity disruption.”<br><br>“Typically, in tertiary hospitals, equipment like oxygen concentrators, pulse oximeter, and nasal prongs were available. However, the number of	Bjorklund 2019: “All six patients who ran out of oxygen survived.”<br><br>“SEAL-bCPAP was designed to not rely on electricity or require intensive monitoring and cost about US$5”<br><br>Farré 2019: “Also, the setting can be simplified if it is used in healthcare centers without oxygen availability (the majority of which are found in rural areas) because one of the rotameters can be excluded, still providing a realistically feasible rescue CPAP setting.”	“Whatever device we are using reliable electricity supply is needed:<br>- to run the bCPAP device<br>- to provide energy of light, monitors, cold chain<br>- also PSA plants needed to provide piped wall O2 or to fill O2-cylinders need electricity to work” (Survey #2, Pediatric Critical Care Physician/Researcher, Sub-Saharan Africa)<br><br>“I would go further in the formulation: Without reliable electricity systems and/or oxygen back-up options (e.g., O2-cylinders) bCPAP cannot be used safely.” (Survey #1, Pediatric Critical Care Physician/Researcher, Sub-Saharan Africa)	

available oxygen concentrators was not adequate to put every child admitted with severe pneumonia due to a high caseload in these settings.”<br><br>“The duration of electric power interruption was different; sometimes it took a few minutes and sometimes longer. The chance of failure of both the power sources (electricity and the generator) was rare, but occurred a few times. For instance, clinicians of Butajira hospital experienced a power cut problem in the pediatric ward for two hours and the automatic power generator was not working that night. In this situation, the patient had to be switched from oxygen concentrator to oxygen cylinder. Two caregivers reported delayed oxygen support for their children during the power failure problem due to mal-functioning of available oxygen cylinders.”<br><br>McCollum 2017: “Another consideration is how to appropriately balance oxygen and CPAP treatments so that treating one child with CPAP doesn’t prevent another child receiving oxygen. One oxygen concentrator provides lowflow oxygen for five to ten children simultaneously but can only serve one child if used for bCPAP.”<br><br>Smith 2017: “Electricity can be intermittent in Salima, Malawi. Our study installed a back-up electrical generator to assure uninterrupted power to the bCPAP devices and oxygen concentrators. While using a generator may decrease 36eneralizability to district hospitals without back-up power supply, assuring uninterrupted power decreases the likelihood that electricity interruptions will become a confounding variable in evaluating bCPAP’s efficacy.”<br><br>Myers 2019: “Approximately eight oxygen concentrators, backed up with oxygen cylinders, were available.”<br><br>Nyondo-Mipando 2020: “Human resource limitations were further exacerbated by constraints around medical equipment and infrastructure, especially lack of electricity […]”			

Wilson 2017: "Both sites had hospital-wide generators that were used during prolonged power outages if fuel was available. The study provided smaller generators to each emergency ward to provide continuous power."<br><br>Buys 2023: "[…] ensuring reliable electricity back-up and maintenance arrangements in order to guarantee the safe function of these devices."<br><br>Chisti 2023: "The opportunities were the availability of oxygen concentrators (with back-up oxygen cylinder provided by the investigators) and automatic power generator back-up in ICMH."<br><br>PATH 2021: "Although some physical nonelectric devices have been developed, most commercially available models are electric and are thus the primary focus of this evidence review."			

CMOC 6. In settings without ICU capabilities (C), creating a system of safe referral to a higher level of care, where available, for patients on bCPAP (R) allows HCWs [a] to seek external support when necessary for sicker patients (M), improving fidelity to clinical indications for bCPAP use (O).

Supporting evidence	Refuting evidence	Supporting quotes	Refuting quotes
Baiden 2021: "In most parts of Ghana, unless a child who presents with acute respiratory distress at a district hospital is well-resuscitated, there is a high probability that the child will die before reaching a regional referral hospital."<br><br>Gebre 2022: "The provision of mechanical ventilation for hypoxemic children in developing countries through endotracheal intubation is not available, feasible, or affordable, except in certain referral hospitals"<br><br>Pulsan 2019: "Our study also confirms a limit to the role of CPAP, while potentially valuable for many children with moderately severe ALRI, without mechanical ventilation and intensive care some children with severe respiratory failure will not be saved by CPAP alone. This is		"Maybe we can add that if referral isn't possible then finding a mechanism to seek help from other resources (PICU/physician/Tele-ICU support) can be one option and should be explored." (Survey #2, Researcher, Pakistan)<br><br>"Do we need to consider time sensitivity on when to mobilise the referral process (sooner rather than later, where available)? - would also improve safety perhaps." (Focus Group #1, Physical Therapist/Researcher, South Africa)<br><br>"In practice I observe clinically very severe children, for whom bCPAP would not be the ideal support even to await external assistance" (Survey #1, Physical Therapist/Researcher, Brazil)	

not to downplay the benefit of using CPAP, but to introduce a sense of realism and avoid vertical thinking that might lead people to believe that CPAP is the answer to pneumonia mortality."<br><br>Buys 2023: "Our facility has a wide drainage area, and optimising referrals is part ofobtaining good outcomes."<br><br>Chisti 2023: "Patients with severe comorbidities were referred to other nearest tertiary hospitals."			

CMOC 7. In settings with limited medical doctor presence (C), team communication and supportive supervision (R) enable bedside HCWs [a] to make time-sensitive decisions and foster collaborative rather than hierarchical interdisciplinary care delivery (M), improving feasibility and fidelity (O).

**Supporting evidence**	**Refuting evidence**	**Supporting quotes**	**Refuting quotes**
Baiden 2021: "This study also demonstrated that physician-supervised nurses could successfully use CPAP in semi-rural district hospitals."<br><br>"Additionally, while the children enrolled in the trials in Ghana and Bangladesh were managed by physician-supervised nurses, those enrolled in the trial in Malawi were managed by clinicians and nurses who worked "without daily physician supervision".14 It is evident that although the trial was reportedly designed to reflect "real world" district hospital settings in Malawi, it enrolled high-risk children who needed care beyond what CPAP is physiomechanically designed to do." [In reference to McCollum 2019 study]<br><br>Duke 2014: "the purpose of this paper is to describe simple CPAP systems that can be used safely and effectively by properly trained nursing staff in a general children's ward or a special care nursery."	McCollum 2019: "[…] bCPAP treatment in a paediatric ward without daily physician supervision did not reduce hospital mortality among high-risk Malawian children with severe pneumonia, compared with oxygen […]"<br><br>Gebre 2022: "Moreover, the treatment given to the Malawian study children was not monitored or supervised by a physician/pediatrician. This feature prompted the recommendation by the Ethiopian Food and Drug Authority (EFDA) in Ethiopia that all of the study hospitals selected for our study not only had physicians administering care, but also supervision by a pediatrician, and that all study subjects be managed in a dedicated room/corner under direct vigilance of the nurses in the pediatric ward."<br><br>"If the results of this effectiveness trial on the clinical use of locally made low-cost BCPAP is found to be beneficial, it could be implemented in other developing country settings where the supervision and routine	"I think the introduction of a middle grade […] [of clinical staff] can develop tremendously good clinical capacities." (Focus Group #2, Pediatric Critical Care Physician/Researcher, Sub-Saharan Africa)<br><br>"In Brazil, we have protocols in most hospitals where supervision of all respiratory support methods is carried out by physical therapists." (Focus Group #2, Physical Therapist/Researcher, Brazil)<br><br>"To successfully treat these patients, it needs more [than protocols]. And it needs […] the training and empowering to have good clinical reasoning. And that needs backup, supportive supervision, possibilities to ask a friend." (Focus Group #1, Pediatric Critical Care Physician/Researcher, Sub-Saharan Africa)	"Empowerment has so much to do with confidence and a sense of authority - which would really challenge the hierarchy of roles." (Survey #2, Pediatric Nurse Practitioner, Malawi)

Nyondo-Mipando 2020: "Medical hierarchy, including the roles and responsibilities of different cadres, sometimes complicated decision-making. While nurses were often the recipients of training as direct users of the system, clinicians held higher authority in decision-making. For example, a nurse from the central hospital narrated a scenario, citing difficulties especially with new clinicians: 'We said that the child was not supposed to be put on CPAP because of the cardiac problem. . . so we discussed [it] with doctors but they insisted to put the baby on CPAP, so the baby was put on CPAP and the condition was still worsening. . . [and] eventually the baby died. . .. It becomes so hard for us to argue with them since they are doctors and they are above us because the moment you say we do not do these things like that, they think that we are underrating them, so these doctors need to be trained more. . . for example, the registrars and consultants'"<br><br>"Even when nurses were trained and encouraged to make the decision to initiate bubble CPAP, some nurses reported reluctance for fear that the clinician would question their decision afterwards."<br><br>Olayo 2019: "Given the shortage of physicians in many LMIC, nurse driven protocols are the most feasible. Empowering non-physician healthcare providers such as nurses and clinical officers to initiate CPAP independently of a physician's order using specific guidelines may expedite placement of the potentially life-saving device on children suffering from acute respiratory distress."<br><br>Wilson 2017: "Since physicians are not usually present in the emergency ward	monitoring of the patients by physicians headed by a pediatrician are available."	"We all […] agree on […] the importance of having in person or via telemedicine a consultant." (Focus Group #1, Pediatric Researcher, Uruguay)<br><br>"Having experienced nurses, nurse practitioners, clinical officers who are familiar with most scenarios and could ask maybe for specific information and advice if needed. And if that ongoing supportive supervision will stay in partnership, that could work." (Focus Group #1, Pediatric Critical Care Physician/Researcher, Sub-Saharan Africa)	

at the study hospitals, emergency ward nurses were responsible for the initiation and management of CPAP."<br><br>PATH 2021: "One study also noted that bCPAP training needs were different between secondary and tertiary levels, with nursing staff at secondary levels being much more reliant on training aides and decision guides than at tertiary levels, where pediatric specialist staff were more common"			

CMOC 8. In settings with adequate staffing (C), assignment of staff to bCPAP patients based on census and acuity (R) allows staff to appropriately allot time for bCPAP setup, monitoring, and adjustments (M), leading to improved fidelity (O).

**Supporting evidence**	**Refuting evidence**	**Supporting quotes**	**Refuting quotes**
André von-Arnim 2017: "LMIC ICUs appear to have higher patient to medical staff ratios, with decreased patient monitoring frequencies, suggesting patient safety should be a focus during the introduction of new respiratory support devices and practices."<br><br>McCollum 2019: "Following initiation, respiratory support for both groups was weaned in 24 h increments based on stepwise procedures published previously. We established 24 h weaning procedures based on our initial observations that frequent bCPAP pressure titration was not feasible in this setting." [In this randomized controlled trial, bCPAP was associated with higher mortality compared to low-flow oxygen.]<br><br>Walk 2016: "Firstly, bCPAP nasal prongs required frequent adjustment to maintain an adequate nasal seal on children owing to dislodgement or mucous occlusion of the prongs. This increased the nursing workload and limited the number of bCPAP set-ups which could be managed at any one time."<br><br>Gulla 2021: "Apart from initiation, other important aspect is to closely monitor and identify early failure within 1-2 hours of initiation and step up the respiratory support in a timely fashion to improve outcome. In LMICs, where the nursing staff to patient ratio is often inadequate, early identification of failure poses an important challenge."<br><br>Buys 2023: "In order to provide bCPAP in this safe and effective manner, the HCUs do require		"There is no doubt about this that there should be adequate staff, and that's obviously going to improve the safety. [….] However, in places where this may be a constraint, the emphasis should be more on maybe training of the existing staff or the caregivers so that there's kind of a participation in the monitoring and use of bCPAP." (Focus Group #3, Clinician/Researcher, India)<br><br>"The work that requires continuous adjustment of the interface used is in accordance with what has been expressed."<br>(Survey #2, Physician/Researcher, Chile)<br><br>"My main concern with this statement is the availability of adequate staff in LMIC. With poor staffing ratios, allocating separate staff for setting up and monitoring bCPAP may not be practically feasible. Training of existing staff is extremely important. This is a practical difficulty." (Survey #2, Clinician/Researcher, India)<br><br>"I do not think it is simply a matter of having 'sufficient' staff assigned, but rather assigning staff trained in bCPAP management […]" (Survey #1, Physical Therapist/Researcher, South Africa)<br><br>"Time for staff is one component of this process, but what is missing in my view is the element of training […]" (Survey #1, Pediatric Pulmonologist/Researcher, South Africa)	

adequate numbers of suitably trained nursing staff."<br><br>Chisti 2023: "The structural and functional challenges in introducing bubble CPAP […] include […] high patient load with an inadequate number of hospital staff […]."<br><br>PATH 2021: "Procedures requiring bCPAPs generally require frequent attention/supervision from clinical staff […]"			

CMOC 9. Comprehensive, contextualized, ongoing, and hands-on clinical and technical staff bCPAP training followed by demonstration of competency in real-world scenarios (R) facilitates retention of knowledge and skills and emergence of local champions (M), particularly in settings without prior bCPAP experience and/or high staff turnover (C), resulting in improved feasibility and fidelity (O).

**Supporting evidence**	**Refuting evidence**	**Supporting quotes**	**Refuting quotes**
André von-Arnim 2017: "The factors most frequently perceived to be very important in promoting the ongoing use of new equipment included equipment safety, adequate training and support of doctors and nurses, and scientific (published) clinical evidence supportive of the intervention."<br><br>Baiden 2021: "They found that nurses who were trained in formal 4-hour sessions at the beginning of the trial retained good skills and knowledge. They performed better on assessment than nurses who had been trained through informal approaches."<br><br>"While all the staff trained in Kintampo hospital were still at post, four out of the five staff trained at the Mampong Hospital had been transferred to other health facilities."<br><br>"In Kenya, CPAP was successfully introduced and sustained in ten government hospitals using a two-day training-of-trainers curriculum. The CPAP training curriculum has been transferred and incorporated into the continuous professional training program of the Kenya Paediatric Association."<br><br>Morris 2014: "One nurse at each hospital study site, chosen as a "team leader," underwent	McCollum 2019: "To optimise protocol adherence, we trained staff on procedures before the study over a 5-day period, including the physiology, application, and monitoring of bCPAP, did twice-annual refresher courses, and provided on-site paediatrician supervision once every 2 weeks. During training and supervisory visits, clinical staff were evaluated on their clinical evaluation of patients, use of decision algorithms, and decision making during directly observed care provision. Remediation took place as necessary." [In this randomized controlled trial, bCPAP was associated with higher mortality compared to low-flow oxygen.]	"I don't believe formal bcpap training alone facilitates retention of knowledge, but rather acquisition of knowledge, perhaps increased competency and confidence in the skill. In my experience, facilitation of knowledge retention occurs with repeated training, follow-up, use of the skill in the practical setting, hands-on skill check off, etc..." (Survey #2, Pediatric Nurse Practitioner, Malawi)<br><br>"They need to be trained. And it's not only bubble CPAP specific. Let's say it's this essential pediatric critical care training. So they need to be able to use the bubble CPAP, they need to use oxygen bag mask, ventilation, treat shock, recognize neurological emergencies, and so forth." (Focus Group #1, Pediatric Critical Care Physician/Researcher, Sub-Saharan Africa)<br><br>"[...] the training shouldn't be just ticking the box. We did some training it. It really needs to be that people colleagues need to demonstrate competencies in simulation training. I think that's always very useful, but then also translating it to clinical realities. And this needs some coaching or accompaniment for a period of time." (Focus Group #1, Pediatric Critical Care Physician/Researcher, Sub-Saharan Africa)<br><br>"I've always often as a trainer, you know, walked out of the room thinking that the trainees had absorbed material. And then, during the practical sessions, you know, making that transition from theoretical to practical can sometimes	"The management of critically ill patients involves more solid training that makes learning and adhering to the protocol more complex. It seems to me that training should be simple and deeply focused on monitoring respiratory deterioration and investigating pathologies or conditions that put the success of CPAP therapy at risk." (Survey #2, Physician Researcher, Chile)

additional training as a superuser."<br><br>"Nonetheless, when a study investigator visited the study sites approximately a year after the study ended, CPAP use seemed to have fallen, and many nurses who were new to the emergency ward reported informally that they were not comfortable using CPAP. Thus, our training program failed to a significant degree. A specific weakness of our study was a failure to work with the local nurses to develop a formal plan for training new personnel and evaluating competence."<br><br>"The proper use of medical devices requires not only knowledge of when to use the intervention but also specific training on how to use, maintain, and repair the device itself, resulting in potential for a substantial efficacy-effectiveness gap"<br><br>Duke 2014: "Introducing any method of CPAP is appropriate only when reliable systems for giving and monitoring oxygen therapy are in place, health workers are adequately trained and close monitoring is assured."<br><br>Gebre 2022: "Other clinicians, who were not involved in this study and did not receive training on bCPAP, were not cooperative in providing the necessary support to the study clinicians during follow-ups ofthe patients with bCPAP in absence ofstudy clinicians in the ward. Therefore, study clinicians felt that before the implementation ofthe new bCPAP in other general hospitals, all staff in the pediatric department need to be trained."<br><br>Gulla 2021: "Training of health care personnel (doctors, nursing staff, technicians) is equally important for successful outcome of non-		be harder for some participants than others, and so really demonstrating that with simulation." (Focus Group #1, Pediatric Pulmonologist/Researcher, South Africa)<br><br>"[The training type] depends [on] the context as well as the level of opportunities available. And they also that turn over the nurses and physicians get that context." (Focus Group #1, Pediatric Respiratory Physician/Researcher, Bangladesh)<br><br>"Once we train someone, we also empower the person to be a trainer." (Focus Group #1, Physician Researcher, Ghana)<br><br>"At the same time, we need to train technicians to guarantee a maintenance system." (Focus Group #1, Pediatric Critical Care Physician/Researcher, Sub-Saharan Africa)	

invasive ventilation in intensive care."<br><br>Myers 2019: "Good nursing care and adequate nurse to patient ratios in a HDU setting are essential in order to reduce complication rates and achieve good outcomes in critically ill children treated with bCPAP. Efficient and sustained training programmes need to accompany the introduction of bCPAP"<br><br>Nyondo-Mipando 2020: "They wished for comprehensive and regular training that provided consistent information on how the CPAP system worked, indications/contra-indications, process of initiation, monitoring and weaning, troubleshooting as well as hands-on skills practice was considered to facilitate its use."<br><br>Olayo 2019: "[…] the training-of-trainers curriculum was successful in transferring skills and knowledge of CPAP between first- and second-generation healthcare providers in a low-income country"<br><br>Pulsan 2019: "Nurses and doctors were trained in how to use the bubble CPAP machine, indications for using CPAP, administering CPAP with required flow rate and oxygen, monitoring of children on CPAP and care of CPAP machines including disinfecting the machine and circuits. Training included the identifying the type of illness and hypoxaemia, the concentrators and how they worked, CPAP equipment and connecting the parts and rectifying equipment malfunctions."<br><br>Wilson 2017: "Every 6 months, the local research staff received refresher training on the use of CPAP and the management of critically ill children"			

Wilson 2017: "Awareness of cultural norms is essential to develop training programs with local staff. In some countries, instructor candidates are accustomed to receiving compensation for traveling to and attending training sessions, so this must be accounted for in the program's budget. Many instructor candidates felt strongly that a training certificate should be received after completing the training session"<br><br>Wilson 2014: "Protocolization of the training program, in conjunction with skills and knowledge assessment, may improve acquisition and retention among second- and future-generation trainees."<br><br>Wilson 2013: "Nasal bubble CPAP was introduced to local nurses through an intensive 4-hour didactic and hands-on training session led by 2 experienced neonatal intensive care unit nurses and a pediatric critical care physician from Columbia University […]"<br><br>Buys 2023: "The study was preceded by the introduction, in 2012, of iterative cycles of training for nurses and doctors regarding the use of nasal prong bCPAP, and the procurement of standardised equipment for use at our facility"<br><br>Chisti 2023: "Before the start of screening and enrolment, the study staff organized a training session for the hospital medical staff (physicians, nurses) at both sites."<br><br>"We also had refresher training to overcome the rapid turnover of the trained doctors and nurses in the paediatric ward […]"<br><br>Path 2021: "Overall lack of training was also cited as a key barrier, exacerbated by high			

staffing turnover and device proliferation."			

CMOC 10. In non-ICU settings (C), designating a specific care area for patients requiring bCPAP (R) lowers HCW[a] cognitive load by enhancing physically clustering of patients requiring similar monitoring (M), improving feasibility (O).

**Supporting evidence**	**Refuting evidence**	**Supporting quotes**	**Refuting quotes**
Duke 2014: "When CPAP is used in a general children's ward, it should be undertaken in a high-dependency area of the ward which should be near the nursing station for close monitoring."<br><br>Gebre 2022: "Moreover, the treatment given to the Malawian study children was not monitored or supervised by a physician/pediatrician. This feature prompted the recommendation by the Ethiopian Food and Drug Authority (EFDA) in Ethiopia that all of the study hospitals selected for our study not only had physicians administering care, but also supervision by a pediatrician, and that all study subjects be managed in a dedicated room/corner under direct vigilance of the nurses in the pediatric ward."<br><br>McCollum 2019: "The use of bCPAP within certain patient populations and non-intensive care settings might carry risk that was not previously 45ealizatio. bCPAP in LMICs needs further evaluation before wider implementation for child pneumonia care."<br><br>"Staff did vital signs and a focused respiratory assessment of children at least every 6 h, including inspecting the nasal interface for patency. Staff did additional evaluations and interventions following standard of care for Malawi district hospitals." [In this randomized controlled trial, bCPAP was associated with higher mortality compared to low-flow oxygen.]<br><br>Myers 2019: "[…] closer observation of selected critically ill children could be provided (spot-checks of vital signs approximately 2–4 hourly)."		"We identified them for a dedicated corner in front of nursing station that those patients should be in very good vigilance in front of trained nurses and that magnificently worked both in Bangladesh and Ethiopia." (Focus Group #3, Pediatric Respiratory Physician, Bangladesh)<br><br>"You don't need a high tech, separate space. I see it more as clustering of similar patients […] for close monitoring, which is there. [….] It's about monitoring and its timely […] and appropriate response." (Focus Group #3, Physical Therapist/Researcher, South Africa)<br><br>"I wholeheartedly support this statement but have concerns about feasibility of such a practice in areas that are constrained with the amount of physical patient care space they have. In extremely busy wards with ~100 children and only 3 staff, this might not work out as planned." (Survey #3, Pediatric Nurse Practitioner, Malawi)<br><br>"I think this only works if you have dedicated and trained staff in the appropriate provider-to-patient ratio." (Survey #2, Pediatric Pulmonologist/Researcher, South Africa)<br><br>"I would even go further and suggest that essential elements of patient circuits and hospital logistics need to be established before starting the use of bCPAP. Examples for key elements of a patient circuit: triage area, emergency zone/department, essential critical care unit or [high dependency unit] setting, step-	

Buys 2023: "PICU space is always at a premium in most low resourced health systems. It is this pressure that led to the realization that something different needed to be done in resource-limited settings to effectively manage children with acute cardiorespiratory distress in high care units outside of PICU where the staff-to-patient ratios fall well below those within intensive care units."<br><br>Chisti 2023: "Those who received bCPAP oxygen therapy were treated in a dedicated corner under nursing vigilance supervised by physicians in the paediatric wards"<br><br>"For the instant visibility of the study patients, the investigators ensured the availability of beds in a dedicated space in front of nursing station that ultimately helped routine follow-ups of the patients by the nurses and supervision by the physicians. This arrangement of beds in front of the nursing station was helpful in a hospital with a high patient load as the KGH, which ultimately helped ensure the availability of beds and oxygen therapy, and the continuous monitoring of patients in a crowded environment."		down unit." (Survey #1, Pediatric Critical Care Physician/Researcher, Sub-Saharan Africa)<br><br>"Cohorting children requiring bCPAP into separate room/cubicle/ or set of beds (in direct visibility) alerts nurses, improves nurse monitoring and effectiveness and safety." (Survey #1, Pediatric Emergency Physician, India)	

CMOC 11. In settings where a caregiver is present at the bedside (C), caregiver education and HCW[a] training in responding to caregiver concerns (R) allow caregivers to feel empowered and psychologically safe to alert HCWs[a] of danger signs and assist in their child's care (M), reducing adverse events and improving fidelity (O).

**Supporting evidence**	**Refuting evidence**	**Supporting quotes**	**Refuting quotes**
Myers 2019: "Caretakers should be integrated in the care and should be encouraged to notify any concern regarding the condition of their child or any malfunctioning equipment."<br><br>Nyondo-Mipando 2020: "Clinicians and nurses interviewed spoke about the need to counsel caregivers and get their consent before initiating CPAP. In reality, time constraints of healthcare providers and difficulties	Gebre 2022: "A physician opined that sometimes caregivers were moving the water bottle to different positions as they required moving their children in their lap and during this process water inside the bottle poured out through the hole on the top of the bottle. Therefore, the depth of water inside the bottle decreased and affected the pressure coming out of the bottle. So, clinicians used to advise caregivers to seek	"Caregivers are always *allowed* to be at bedside -- but their involvement is often limited by their own comfort level and confidence to participate. Educating caregivers increases the likelihood that they will join in their child's care." (Survey #2, Pediatric Nurse Practitioner, Malawi)<br><br>"What we have done in Bangladesh and Ethiopia, we educated the mother that if the mother can see [nasal	

explaining CPAP in lay language at times lead to inadequate explanations for caregivers.”<br><br>Sessions 2020: “The majority of mothers preferred information about the treatment to be given before any medical care was provided to the child, as was done in this trial, to address any concerns or allow the mother to decline treatment.”<br><br>“Caregivers displayed a strong support for both oxygen and bCPAP following treatment, even if their child died. This suggests that with exposure and education both can be considered acceptable therapies among caregivers.”<br><br>Walk 2016: “One alternative solution could be to train patient carers to assist nurses with simple but frequently required respiratory support tasks such as maintaining an adequate nasal prong seal.”<br><br>Chisti 2023: “There was initial anxiety of few mothers due to initial crying, dryness of the mouth & lips of their children; however, eventually all the mothers became happy with the positive outcome (calm, relaxed, breathing comfortably, sleeping, opening of eyes, smiling, improved oxygen saturation, faster disappearance of grunting sound/other danger signs, able to feed, shorter hospital stay) with faster hospital discharge that helped to reduce caregivers’ direct and indirect cost of purchasing food, medicines and traveling to the hospitals”	help to solve such incidents like bottle fall and water splash.”<br><br>Myers 2019: “One parent refused the continuation of bCPAP.”<br><br>Sessions 2020: “The intermittent removal of the mask or nasal prongs by caregivers during oxygen and bCPAP treatments could interfere with treatment effectiveness and lead to poorer than expected outcomes. Caregivers implied that removal of oxygen and bCPAP treatments occurred when healthcare providers were not present, perhaps while caring for other children or at night when the staff- to-patient ratio was lower. While the frequency of removal was unclear, its occurrence is nevertheless alarming.”	secretions], mother can ask the nurses, even if the nurse might not be available at that time, and it worked dramatically. Initially, they were hesitant, but eventually they responded very well.” (Focus Group #1, Pediatric Respiratory Physician, Bangladesh)<br><br>“We should […] also train the trainers to listen to parents and to help parents.” (Focus Group #1, Pediatric Researcher, Uruguay)<br><br>“That capacity building curriculum […] for caregivers could mean […] recognition of danger signs, understanding of clinical interventions, […] care support, like positioning, […] and other elements. But we need to realize that potential post-discharge mortality of the children we talk about can be very high. So that capacity building of parents can then also be linked to discharge preparation and follow up.” (Focus Group #1, Pediatric Critical Care Physician/Researcher, Sub-Saharan Africa)	

CMOC 12. In settings where a caregiver is present at the bedside (C), explanation to caregivers regarding bCPAP before therapy initiation (R) decreases caregiver distress and increases caregiver acceptance of bCPAP (M), decreasing caregiver-initiated disruptions in care and enhancing fidelity (O).

Supporting evidence	Refuting evidence	Supporting quotes	Refuting quotes
Gebre 2022: A physician opined that sometimes caregivers were moving the			

water bottle to different positions as they required moving their children in their lap and during this process water inside the bottle poured out through the hole on the top ofthe bottle. Therefore, the depth ofwater inside the bottle decreased and affected the pressure coming out ofthe bottle. So, clinicians used to advise caregivers to seek help to solve such incidents like bottle fall and water splash.<br><br>Gondwe 2017: "In this study, the caregivers who found their infants already commenced on bCPAP were more stressed than their counterparts."<br><br>Nyondo-Mipando 2020: "Study participants reported that caregivers sometimes had fears that […]—a perception that may have been influenced by the lack of clear, effective communication between providers and caregivers."<br><br>Sessions 2020: "Mothers often reported being told to remove the equipment temporarily or permanently by other caregivers and this led to several self-reports of interrupting care."			

CMOC 13. In high-mortality settings where community members may associate oxygen therapy with death (C), community education and outreach (R) reduce community fears related to inpatient care and oxygen therapy (M), improving timeliness of care-seeking, community acceptance of bCPAP, and fidelity (O).

**Supporting evidence**	**Refuting evidence**	**Supporting quotes**	**Refuting quotes**
<u>Sessions 2020:</u> "Community perceptions of oxygen and bCPAP were widely negative. Mothers recounted that they are told that 'oxygen kills babies'. They are often fearful of allowing their child to receive oxygen therapy and will delay treatment or seek alternative therapies."<br><br>"Almost all information participants received from the community was negative. Eighty- three per cent of mothers (45/54) explicitly, and			

without prompting, mentioned hearing negative opinions of supplemental oxygen therapy in the community, many using the phrasing that 'oxygen kills'"<br><br>"The knowledge about bCPAP was significantly lower; most reported they had never heard of bCPAP before being educated by the study staff at the hospital. However, those who had heard of bCPAP reported similar negative concern that it can worsen the child's condition or kill the child."<br><br>"Another common theme was the desire for education in the communities. Mothers recognised a need for community sensitisation to feel more comfortable about coming to the hospital."<br><br><u>Nyondo-Mipando 2020</u>: "Study participants reported that caregivers sometimes had fears that the many tubes interfered with breathing and that oxygen therapy was associated with death […]"<br><br><u>Gondwe 2017</u>: "Perceptions about bCPAP treatment varied, but common to most participants was the fear of injury to or death of their child when bCPAP was initiated."<br><br><u>Morris 2014</u>: "We did not build a process of community engagement or consultation with community leadership into our study, but ethics review boards should consider whether such engagement is warranted for individual studies."<br><br><u>WHO 2016</u>: "Parents must be educated about the need for oxygen in order to alleviate their fears. Many parents are afraid of oxygen and oxygen catheters, perhaps because they have seen other children receive oxygen just before they died and may fear that it			

was the oxygen that caused the death."			

CMOC 14. In settings with prior adoption of basic respiratory care practices (C), integration of bCPAP within existing functional respiratory care services (R) results in HCWs[a] perceiving bCPAP as an extension of existing care services (M), improving adherence to intended protocols and fidelity (O).

Supporting evidence	Refuting evidence	Quotes
Morris 2014: "A decision to introduce a new acute care medical device may require particular scrutiny, especially in settings where health care capacity may not be sufficient to deliver even basic medical care."<br><br>Duke 2014: "Introducing any method of CPAP is appropriate only when reliable systems for giving and monitoring oxygen therapy are in place, health workers are adequately trained and close monitoring is assured."<br><br>Gebre 2022: "This study findings are also consistent with the previous study that reported medical equipment was often broken, or inappropriate for use [24]. Therefore, we suggest that ensuring the availability, functionality, good quality and proper maintenances of the existing medical equipment of the hospitals is very important for the successful implementation of the bCPAP treatment."<br><br>Kortz 2017: "Treatment for severe paediatric pneumonia ideally includes six elements: provider knowledge to appropriately manage pneumonia; oxygen; antibiotics; non-invasive positive pressure ventilation (such as bCPAP); non-invasive monitoring (continuous pulse oximetry); and nasopharyngeal (NP) suctioning."		"Yes oxygen systems provide a framework, but CPAP is unique enough that the resource utilization is much different." (Survey #1, Pediatric Pulmonologist/Researcher, South Africa)

André von-Arnim: "To ensure successful translation of technology research into medical practice, data on human factors and device environment are important. In the case of pediatric respiratory equipment these include facility infrastructure and staffing, commonly available equipment and expertise levels."<br><br>Chisti 2023: "Due to an initial insufficiency and/or non-functionality of medical equipment, the study team solved most of these challenges by making pulse oximeters, oxygen concentrators, suction machine, and nebulisers available before the introduction of bubble CPAP oxygen therapy in both the study hospitals."		

CMOC 15. In settings with experience successfully adapting guideline-recommended practices to local context using a quality improvement approach (C), evidence-based, locally relevant bCPAP clinical use guidelines (R) normalize intended bCPAP care practices (M), improving fidelity (O).

Supporting evidence	Refuting evidence	Supporting quotes	Refuting quotes
André von-Arnim 2017: "Most responders indicated that their primary work facility had successfully implemented standardized clinical protocols for management of common acute illness. Methods most helpful for implementation of standardized clinical protocols included adequate training and introduction to the new practice, ongoing support for the practice change, and availability of all necessary equipment."<br><br>"To have global impact, it is not sufficient to simply develop an affordable, effective respiratory support system. In addition, staff must be trained to deliver the therapy, clinical guidelines and monitoring for use must be in place"<br><br>Browde 2019: "At RCWMCH, institutional guidelines recommend the use of nCPAP or HFNC, using a stand-alone Bubble-nCPAP device (Fisher & Paykel Healthcare, New Zealand) as the standard of care		"Another important consideration is […] monitoring the outcomes after bCPAP program start and sharing with all users leading to encouragement and also modifications as per learnings." (Survey #2, Researcher, Pakistan)	

in children presenting in severe respiratory distress."<br><br>Duke 2014: "Implementation requires procurement expertise, standardisation, uniformity and compatibility of equipment, maintenance guidelines and basic engineering capacity, and clinical guidelines on how to care for children with severe respiratory infection and neonates with respiratory distress, including when to use oxygen and when to use CPAP."<br><br>Chisti 2023: "Furthermore, a detailed and simple training manual and standard operation procedures (SOPs) in English and local language (Bangla) on the implementation of bCPAP oxygen therapy and the maintenance of medical equipment must available in the treatment area."			

CMOC 16. In settings with other new initiatives being implemented simultaneously with new bCPAP implementation (C), a coordinated strategy for rolling out multiple programs (R) results in more efficient resource allocation and reduces the impact on HCW[a] cognitive load (M), improving fidelity (O).

Supporting evidence	Refuting evidence	Supporting quotes	Refuting quotes
Sessions 2019: "During peak pneumonia season, the number of patients meeting these criteria can increase to 45 patients per month, which would represent an additional 78 work hours per month on average. Additionally, SDH treats an average of 50 patients per month with WHO-defined severe pneumonia without HIV, malnutrition, or hypoxemia, and up to an average of 94 patients monthly during peak pneumonia season. If all patients with severe pneumonia, regardless of comorbidity, received bCPAP instead of low-flow nasal oxygen therapy health care workers could face an additional 164 h of work per month during peak pneumonia season."		"If you focus it down to the healthcare provider level, it kind of depends on who's doing what within that system, in terms of where people are getting pulled." (Focus Group #2, Pediatric Pulmonologist, South Africa)<br><br>"My thinking is that multiple initiatives implemented at the same time could be distracting for clinicians and actually make resource allocation less efficient. But I could also see how appropriately paired initiatives could have a synergistic effect." (Survey #2, Pediatric Nurse Practitioner, Malawi)	"I believe that new technologies should be introduced one at a time and not simultaneously." (Survey #2, Physical Therapist, Brazil)

		If there are training programs […] that pull people out of their workplace, that can be difficult. But let's say if we have more coaching strategy, on the job training where we maybe have a bCPAP initiative but at the same time improve the management of shock, transfusion, and coma/convulsion, that may be complementary and may go together. (Focus Group #2, Pediatric Critical Care Physician, Sub-Saharan Africa)	

CMOC 17. Prior adoption of clinical adverse event prevention strategies (C), when integrated with a bCPAP program (R), enhances HCW [a] competence and confidence in using these strategies with bCPAP (M), reducing bCPAP-related adverse events and enhancing fidelity (O).

**Supporting evidence**	**Refuting evidence**	**Supporting quotes**	**Refuting quotes**
Brown 2020: "This surprising finding was attributed to post-randomisation harm, related to aspiration and pneumothorax in a minimally monitored environment." [In reference to McCollum 2019 paper]<br><br>Chisti 2015: "All children received WHO standard management for very severe pneumonia, including parental ampicillin and gentamicin, nasogastric feeding or intravenous fl uids if the child had very severe respiratory distress, and hourly monitoring of clinical signs of respiratory distress and SpO2"<br><br>Duke 2014: "Infants and children on CPAP in moderate respiratory distress should be fed by nasogastric tube, ideally using expressed breast milk from their mothers. There is some risk of aspiration if fed by mouth, but it is minimal if fed by a well sited nasogastric tube."<br><br>Gulla 2021: "Complications related to NRS are: […] Aspiration: may occur due to		"[I] wonder if another statement should be added recommending training for and successful and safe [nasogastric tube] placement, as evidence suggests improved safety with [nasogastric tube]?" (Survey #1, Physical Therapist/Researcher, South Africa)<br><br>"Body positioning (30 degrees elevation) has been used to avoid the same outcomes […]" (Survey #1, Physical Therapist/Researcher, Brazil)<br><br>"There can be so much trouble associated with gastric tubes that it's a real important element of the training, especially after initial stabilization" (Focus Group #1, Pediatric Critical Care Physician/Researcher, Sub-Saharan Africa)	"Hunger is an irritating factor that aggravates respiratory failure and in this scenario a well-fed infant will be more comfortable receiving nasal CPAP support. In our experience, babies tolerate feeding with this therapy when their respiratory distress evaluation scale is moderate." (Physician/Researcher, Chile)

gastric distension and vomiting"<br><br><u>Larsen 2021</u>: "The infant was breastfeeding during which we paused bCPAP to avoid aspiration. No nasogastric tube was inserted due to lack of space at the nasal interface, so we closely monitored for gastric distension."<br><br><u>McCollum 2019</u>: "Few enrolled children had a nasogastric tube inserted despite nearly all being eligible for nasogastric tube placement. The influence of infrequent nasogastric tube use on these findings is unclear"<br><br>"Of note, although 619 (96%) of 644 children enrolled onto the trial met nasogastric tube insertion criteria, records indicated that only 101 (16%) of 619 participants received one. A 7% lower proportion of bCPAP children (40 (13%) of 308), compared with oxygen children (61 (20%) of 311), were documented to have had a tube inserted (p=0·024)."<br><br>"[…] it is also possible that the combination of higher initiating CPAP pressures, enteral feeding, and low proportion of nasogastric tube use among bCPAP recipients predisposed patients to unrecognised aspiration events. Our findings might not be applicable to settings that have more widely adopted the use of nasogastric tube."<br><br><u>Myers 2019</u>: "Additional aspects of care for patients on bCPAP included head-up positioning, nasogastric tube (NGT) insertion for initial gastric decompression and drainage"<br><br><u>Smith 2017</u>: "Children with any general danger sign, 3 or more respiratory danger signs, or apnoea or grunting in isolation are not allowed to eat by mouth. Instead, these			

children receive a nasal gastric tube for feeding of expressed breast milk or formula every 2 hours at a standardised amount calculated to provide appropriate calories and fluid."<br><br>Wilson 2019: "Over 95% of patients in the Malawi study were eligible for nasogastric tube insertion per the study protocol but only 13% of patients receiving bCPAP were documented as having them placed. Feeding patients orally while in respiratory distress can lead to aspiration events and bCPAP could increase this risk further via gastric distension. Nasogastric feeds bypass the upper airway and might decompress the stomach avoiding further gastric distention. The Ghana study did not allow children to take feeds by mouth while in respiratory distress and most patients in Bangladesh received nasogastric tubes while on bCPAP. Both the Ghana and Bangladesh studies started bCPAP at 5 cm H_2O, whereas the Malawi trial initiated bCPAP at 7 cm H_2O or 8 cm H_2O, possibly increasing the risk of aspiration."			

CMOC 18. In settings in which single-use consumables are reused (C), following a safe and standard protocol for cleaning, disinfection, sterilization, and cessation of reuse (R) results in staff mitigating infection risk and recognizing material degradation (M), reducing adverse events and enhancing fidelity (O).

**Supporting evidence**	**Refuting evidence**	**Supporting quotes**	**Refuting quotes**
Morris 2014: "On a return visit to Ghana, we noted that at least one hospital was washing and reusing interfaces, though their durability and the degree to which they can be disinfected is not known."<br><br>Duke 2014: "Even simple CPAP or oxygen systems carry a risk of hospital-acquired infection if the equipment is not cleaned appropriately. Cleaning should be done after every patient has used the CPAP equipment, and at least weekly if the same child is on CPAP for over a week. A	André von-Arnim 2017: "Reusability, while understood to be prevalent, and anecdotally desirable, was not a key factor for promoting new technology use for survey respondents from LMIC."	"Single - use consumables shouldn't be reused by definition.<br>If reuse in same patient, material degradation and other factors may be considered.<br>Together with the protocol, it may be needled specific areas and equipment for disinfection and sterilization." (Biomedical Engineer, World Health	

spare circuit is essential to replace the one that is being cleaned so that treatment is not interrupted. The CPAP circuit (inspiratory and expiratory limb, bottle and lid and connections) should be thoroughly cleaned."<br><br>McCollum 2019: "Because of budgetary constraints and supply chain issues in Malawi and other LMICs, respiratory supplies are in our experience almost always disinfected and reused during routine care, and we took this approach."<br><br>Bjorklund 2019: "If needed, the apparatus could be cleaned and re-used, except the earplug material which was removed and replaced."<br><br>Wilson 2019: "The authors offer several potential explanations for the increased mortality seen in their specific population: aspiration events, pneumothoraces, nasal airway obstruction, oxygen toxicity, absence of physician supervision, and equipment contamination." [in reference to the McCollum 2019 study]<br><br>Wilson 2014: "The CPAP in Ghana study was begun in full knowledge that single-use nasal prongs are not affordable on an ongoing basis. We found that at least one hospital is washing and reusing prongs to circumvent this problem."		Organization, Survey #3)<br><br>"This is difficult, as design and manufacture of single use products are approved for use based on single-use protocols. Cleaning and reuse can have substantial degradative results on the product, that can lead to material failure." (Biomedical Engineer, USA, Survey #3)<br><br>"Low-cost disinfection which at its heart was a strategy of re-use was a leading focus of a failed commercialization attempt by our team. [….] I'm not sure how to comment here - "protocol for supply cleaning, reuse, and cessation" or something in step two to ensure there is stopping point really comes to mind. The statement as is lets the status quo remain which is save lives at any cost even if you stretch tools to negatively impact quality of life, permanently." (Former CEO of Medical Device Startup, Survey #3)<br><br>"We have the three steps: […] cleaning, disinfection, and then sterilization, depending on the type of consumable. [….] Even if the process or protocol is followed well, there is a lifespan for each of the consumables, so that needs to be considered." (Biomedical Engineer, World Health	

		Organization, Focus Group #3)<br><br>“Local availability of sterilisation, parameters to ascertain adequate sterilisation of circuits and regular checking for wear and tear of circuit are all imp[ortant] considerations.” (Survey #2, Researcher, Pakistan)	
